# Conjugative Plasmids of *Neisseria gonorrhoeae*

**DOI:** 10.1371/journal.pone.0009962

**Published:** 2010-04-01

**Authors:** Emilia Pachulec, Chris van der Does

**Affiliations:** 1 Department of Microbiology, Groningen Biomolecular Sciences and Biotechnology Institute, University of Groningen, Haren, The Netherlands; 2 Zernike Institute for Advanced Materials, University of Groningen, Haren, The Netherlands; 3 Department of Ecophysiology, Max Planck-Institute for Terrestrial Microbiology, Marburg, Germany; National Institutes of Health, United States of America

## Abstract

Many clinical isolates of the human pathogen *Neisseria gonorrhoeae* contain conjugative plasmids. The host range of these plasmids is limited to Neisseria species, but presence of a tetracycline (*tetM*) determinant inserted in several of these plasmids is an important cause of the rapid spread of tetracycline resistance. Previously plasmids with different backbones (Dutch and American type backbones) and with and without different *tetM* determinants (Dutch and American type *tetM* determinants) have been identified. Within the isolates tested, all plasmids with American or Dutch type *tetM* determinants contained a Dutch type plasmid backbone. This demonstrated that *tetM* determinants should not be used to differentiate between conjugal plasmid backbones. The nucleotide sequences of conjugative plasmids with Dutch type plasmid backbones either not containing the *tetM* determinant (pEP5233) or containing Dutch (pEP5289) or American (pEP5050) type *tetM* determinants were determined. Analysis of the backbone sequences showed that they belong to a novel IncP1 subfamily divergent from the IncP1α, β, γ, δ and ε subfamilies. The *tetM* determinants were inserted in a genetic load region found in all these plasmids. Insertion was accompanied by the insertion of a gene with an unknown function, and rearrangement of a toxin/antitoxin gene cluster. The genetic load region contains two toxin/antitoxins of the Zeta/Epsilon toxin/antitoxin family previously only found in Gram positive organisms and the virulence associated protein D of the VapD/VapX toxin/antitoxin family. Remarkably, presence of VapX of pJD1, a small cryptic neisserial plasmid, in the acceptor strain strongly increased the conjugation efficiency, suggesting that it functions as an antitoxin for the conjugative plasmid. The presence of the toxin and antitoxin on different plasmids might explain why the host range of this IncP1 plasmid is limited to *Neisseria* species. The isolated plasmids conjugated efficiently between *N. gonorrhoeae* strains, but did not enhance transfer of a genetic marker.

## Introduction

The obligate human pathogen *Neisseria gonorrhoeae* colonizes mucosal tissues in the urogenital tracts to cause the sexually transmitted disease gonorrhea [1]. Although gonorrhea can still be treated with antibiotics it has progressively accumulated resistance against many antibiotics like e.g. penicillin, ciprofloxacin, tetracycline, azithromycin and cefixime, and currently not many new antibiotics are available [2]. The rapid spread of antibiotic resistance is caused by the high rate of horizontal gene transfer in *N. gonorrhoeae*, resulting in a panmictic population structure [3]. Horizontal gene transfer in *N. gonorrhoeae* is driven by its high rates of natural transformation and recombination. DNA for horizontal gene transfer is most likely derived from lysis, but transfer frequencies are approximately 500 fold increased in strains which secrete DNA via the Type IV secretion system found within the recently characterized Gonococcal Genetic Island (GGI) [4]. Horizontal gene transfer also occurs via conjugative plasmids, which can not only transfer their own DNA, but often can also co-mobilize chromosomal or plasmid DNA. Currently three types of gonoccocal conjugative plasmids have been described in *N.gonorrhoeae*; a 24.5 MDa plasmid with no detectable marker, and two 25.2 MDa plasmids which contain the *tetM* determinant [5].

The 24.5 MDa plasmid (also called pLE2451) was first found in 1974 in the United States in clinical isolates of non-penicillinase and penicillinase producing *N. gonorrhoeae* [6], [7], [8]. The host range of the 24.5 MDa plasmid is limited to *N. gonorrhoeae* and to some strains of *Neisseria cinerea* [9]. The 24.5 MDa plasmid was shown not to be involved in the mobilization of genomic DNA. In 1982, 25.2 MDa conjugative plasmids containing *tetM* determinants were identified in clinical isolates from the United States [10]. *TetM* determinants are transposon-borne determinants found in many organisms like e.g., *Streptococcus* [11], *Mycoplasma hominis* [12], *Ureaplasma urealyticum* [13], *Enterococcus* spp [14] and *Gardnerella vaginalis* [5], [15] and are responsible for high levels of tetracycline resistance. The *tetM* determinants within the 25.2 MDa plasmids are derived from a so-called class II Tn916-like transposon insertion which means that large parts of the Tn916-like transposon are deleted but that the *tetM* determinant is maintained [16]. Nowadays, gonoccocal isolates resistant to high doses of tetracycline (MIC>8 µg/ml) carrying 25.2 MDa plasmids have been isolated worldwide [5], [17], [18], [19]. Restriction endonuclease mapping and Southern blotting of conjugative plasmids from different isolates revealed two different 25.2 MDa conjugative plasmids [18], which were named the “American” and “Dutch” type plasmids [20]. The restriction map of the Dutch type plasmid strongly resembled the restriction map of the 24.5 MDa conjugative plasmid and it was proposed that the Dutch type 25.2 MDa plasmid is a derivative of the 24.5 MDa plasmid by an insertion of the *tetM* determinant [5], [21]. Early studies proposed that the American type plasmid might be similar to both the conjugative and the Dutch type plasmid in areas of conserved functions like replication, incompatibility and transfer function [18], but restriction endonuclease mapping demonstrated large differences between American and Dutch type plasmids [22]. Sequencing of the *tetM* regions of American and Dutch type plasmids also revealed differences within the two *tetM* determinants, and it was proposed that the *tetM* determinant found in the American type conjugative plasmids has a different origin from the *tetM* determinant present in the Dutch type conjugative plasmids [23]. In a different study 13 American *tetM* determinants were linked to the restriction maps of American type conjugative plasmids [20]. Based on the different *tetM* sequences PCR primers were developed which could differentiate between the 2 different *tetM* determinants. The *tetM* determinant has been generally linked to the conjugation plasmid type, although some *tetM* determinants have also been identified in plasmids with restriction maps that differ from the initially identified American and Dutch types, indicating that either the American and Dutch type plasmids are still evolving or that the different *tetM* determinants have been inserted into different families of conjugative plasmids [20]. Unfortunately, it is unclear in many of the studies whether Dutch or American type plasmids were used, and whether differences would be observed between these two types of plasmids. 25.2 MDa plasmids showed a broader host range then the 24.5 MDa plasmid and could be transferred to other *Neisseria* species like *Neisseria cinerea, Neisseria meningitidis, Neisseria mucosa, Neisseria flava, Neisseria lactamica, Neisseria flavescens, Neisseria subflava*, and *Neisseria perfiava* [17]. Plasmids carrying the *tetM* determinant were also identified in tetracycline-resistant *Neisseria meningiditis*, *Kingella denitrifans* and *Eikenella corrodens* while *Neisseria subflava* biovar *perflava*, *Neisseria sicca* and *Neisseria mucosa* isolates carried the *tetM* determinant in the chromosome [24]. The 25.2 MDa conjugative plasmid can also be transfered to *Escherichia coli*, but can not be maintained in this species [25].

The conjugative plasmids were also shown to be involved in the mobilization of small non selftransmissible β-lactamase gonococcal plasmids [26]. Mobilization by the conjugative plasmids was observed after short mating to other *Neisseria* species, to *Haemophilus influenzae* and to restriction-deficient *Escherichia coli* [27]. Mobilization occurs either via the *oriT*-binding MobA mobilization protein [28] or with lower efficiency via co-integration with the conjugative plasmid [25]. Interestingly some plasmids can only be mobilized by a 25.2 MDa conjugative plasmid and not by the 24.5 MDa plasmid [29].

These gonoccocal conjugative plasmids have been studied for many years, and in this paper we demonstrate that within our isolates both Dutch and American type *tetM* determinants are found in plasmids with a Dutch type backbone. The sequence of the Dutch type backbone, and the sequences of the genetic load regions in which the Dutch and American type *tetM* determinants were inserted were determined. Based on these obtained sequences the plasmids were further characterized, and their involvement in DNA transport was compared to the type IV DNA secretion system encoded within the Gonococcal Genetic Island.

## Results

### Isolation and sequencing of a conjugal plasmid with a Dutch type *tetM* derminant

**Figure 1:**
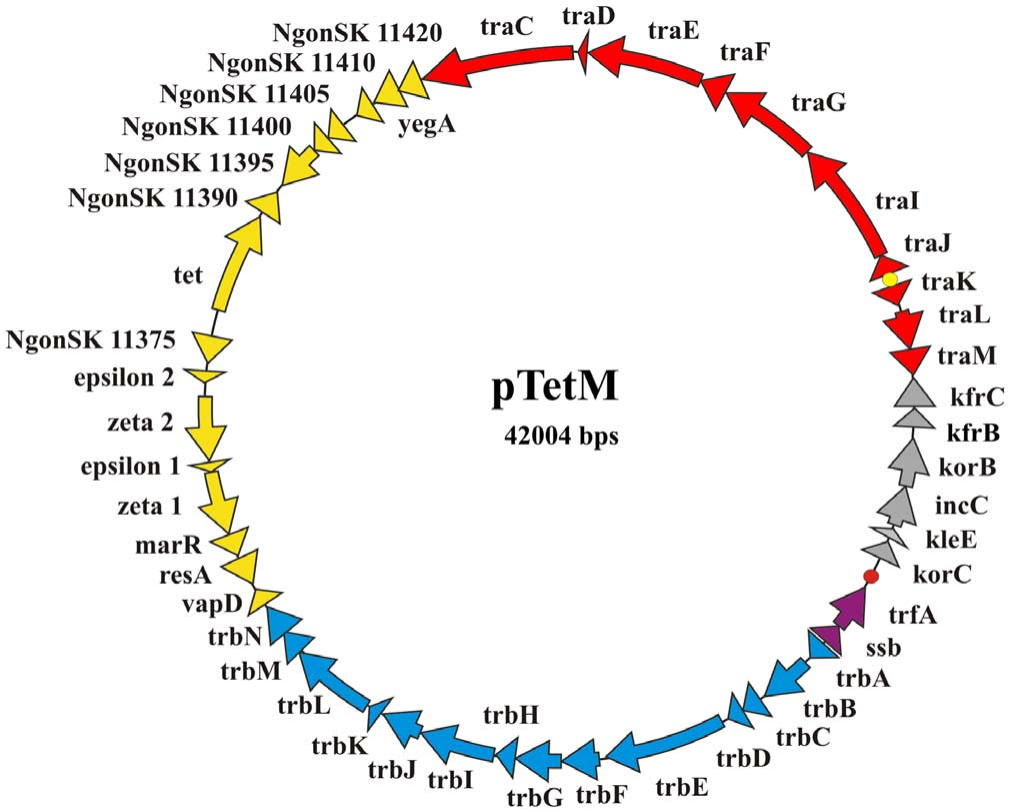
Circular map of the Neisserial conjugative plasmid (pEP5289) with the Dutch type *tetM* determinant and a Dutch type backbone. The open reading frames are colored as follows: red, conjugative transfer (tra); blue, mating pair formation (trb); purple, replication initiation (rep); grey inheritance/control (ctl) and yellow, genetic load. The origin of replication (*oriV*) and the origin of transfer (*oriT*) are indicated with red and yellow circles respectively.

We set-out to study the conjugative plasmids of *N. gonorrhoeae*. A collection of 60 strains from low-passage clinical isolates was obtained from the Municipal Health Service, GG&GD in Amsterdam, The Netherlands. The strains were obtained from the proctum, the cervix, the urethra, and the tonsil. This collection was a selection of strains resistant to the fluoroquinolones: ciprofloxacin and oxacillin, tetracycline, penicillin, and strains resistant to all these antibiotics (see table 1). These strains were tested for the presence of the Dutch and American type *tetM* determinants by two different PCR amplification methods described previously [30], [31]. The results for both PCR methods agreed for all samples. Within 42 isolates tested 6 contained the American type *tetM* determinant and 6 contained the Dutch type *tetM* determinant. As expected, the *tetM* determinants were only detected in the strains with resistance above 1 µg/ml tetracycline. *Neisseria* strain 5289 carrying the Dutch type *tetM* determinant was chosen for plasmid isolation. Analysis of the isolated plasmid by gel electrophoresis identified products running at ∼40 kb, ∼10 kb and ∼4 kb. Both the 40 kb and 10 kb products hybridized with a Dutch type *tetM* probe after Southern blotting, demonstrating that they are two differently coiled forms of the plasmid. The product at ∼4 kb was pJD1, a 4.207-bp cryptic plasmid, found in most *N. gonorrhoeae* strains [32]. To confirm that the isolated plasmid was the Dutch type plasmid, digestion with *Bgl*I was performed and the obtained digestion pattern was similar to the previously observed pattern for a Dutch type plasmid [20]. To test the efficiency of transfer of the plasmid of strain 5289, the plasmid was first conjugated to our laboratory strain MS11, and overnight matings were performed in liquid medium and on filters. The mating efficiency was high (10^−1^ transconjugants/donor) and liquid mating was as efficient as surface mating. The plasmid was sequenced via the shotgun method with at least 6 times coverage followed by gap closure based on PCR amplification. Complete sequencing of the plasmid (pEP5289) resulted in a sequence of 42.004 bp. The plasmid has a G+C content of 48%, compared to 52.7% for *the N. gonorrhoeae* chromosome and 44% for the horizontally acquired Gonococcal Genetic Island. The sequence was identical to contig NZ_ABZP01000196 of *N. gonorrhoeae* strain SK-92-67 obtained during the recent sequencing of 15 different *N. gonorrhoeae* strains. The plasmid contained 3 DNA Uptake Sequences (DUS) which is a 14-fold lower frequency then observed for the *N. gonorrhoeae* chromosome. Manual annotation revealed 48 open reading frames (see table 2). Analysis of the annotated open reading frames, revealed a modular structure typical for the IncP1 plasmids. The plasmid is organized in modules for replication initiation (Rep), conjugative DNA-transfer (Tra), mating-pair-formation (Trb), stable plasmid inheritance and control (Ctl) (see figure 1). Furthermore, the plasmid contains a ‘genetic load’ module containing several genes inserted between the Trb and Tra regions.

**Table 1:** Characteristics of clinical isolates used in this study (S-sensitive).

Isolate nr	Material source	Ciprofloxacin MIC µg/ml	Tetracyclin MIC µg/ml	Penicillin MIC µg/ml	GGI presence	*type of plasmid*
4314	urethra	2	S	S	−	Conjugative
4465	proctum	4	S	S	+	Conjugative
4485	urethra	3	S	S	−	Conjugative
4511	proctum	3	S	S	−	Conjugative
4518	urethra	0.5	S	0.75	+	Conjugative
4603	proctum	6	S	S	−	Conjugative
4890	urethra	1.5	S	S	−	Conjugative
4892	urethra	1	S	S	−	Conjugative
4894	proctum	0.75	S	S	−	Conjugative
5308	urethra	8	S	S	−	Conjugative
4393	proctum	S	1	2	−	American
4703	cervix	S	6	>32	+	Dutch
4731	urethra	S	0.38	S	+	Not detected
5050	urethra	S	64	S	−	American
5135	proctum	S	16	S	+	American
5289	urethra	S	24	S	+	Dutch
5291	cervix	S	>256	S	−	American
5351	urethra	S	24	S	+	Dutch
5371	urethra	S	16	>32	−	Dutch
4290	urethra	S	8	0.125	+	American
4415	tonsil	S	S	3	−	Conjugative
4458	cervix	S	S	12	+	Conjugative
4547	urethra	S	24	8	+	Dutch
4635	urethra	2	S	>32	−	Conjugative
4722	urethra	S	S	2	+	Not detected
4993	urethra	2	S	0.19	+	Conjugative
5224	urethra	S	16	24	+	American
5233	urethra	S	S	6	+	Conjugative
5259	proctum	S	S	0.75	+	Conjugative
5343	urethra	S	8	24	+	Dutch
4305	proctum	S	S	S	+	Not detected
5312	proctum	S	S	S	+	Not detected
4392	tonsil	S	S	S	+	Not detected
4308	urethra	S	S	S	+	Not detected
4745	urethra	S	S	S	+	Conjugative
4818	tonsil	S	S	S	+	Conjugative
5020	urethra	S	S	S	−	Not detected
5065	proctum	S	S	S	+	Not detected
5066	urethra	S	S	S	+	Conjugative
5067	cervix	S	S	S	+	Not detected
5284	urethra	S	S	S	+	Not detected
5375	urethra	S	S	S	+	Not detected

**Table 2:** Annotation of open reading frames located within the neisserial conjugative plasmid (pEP5289) with a Dutch type *tetM* determinant and a Dutch type backbone.

gene	length (bp)	homologue of putative protein	identity/range (aa)	function of homologue
*trbA*	366	TrbA of *Pseudomonas aeruginosa*	50%/116	transcriptional repressor
*trbB*	999	TrbB of *Legionella pneumophila* str. Corby	55%/292	VirB11 like conjugal transfer ATPase (VirB11)
*trbC*	384	TrbC of plasmid pB3	72%/112	Prepilin (putatively circularized by TraF)
*trbD*	318	TrbD of *Xylella fastidiosa*	59%/103	conjugal transfer
*trbE*	2565	TrbE of Birmingham IncP-alpha plasmid	60%/853	VirB4-like conjugal transfer ATPase
*trbF*	708	TrbF of *Azoarcus* sp. EbN1	55%/230	DNA transfer
*trbG*	867	TrbG of *Azoarcus* sp. EbN1	53%/275	VirB9-like Core complex component
*trbH*	411	TrbH of IncP-1 plasmid pKJK5	33%/151	VirB7-like Core complex component
*trbI*	1440	TrbI of *Acidovorax* sp. JS42	46%/477	VirB10-like Core complex component
*trbJ*	777	TrbJ of *Bordetella pertussis*	48%//226	VirB5
*trbK*	141			
*trbL*	1629	TrbL of *Azoarcus* sp. EbN1	46%/215	VirB6-like inner membrane protein
*trbM*	579	TrbM of *Bordetella pertussis*	48%/154	conjugal transfer
*trbN*	600	TrbN of *Sphingomonas* sp. A1	49%/207	lytic transglycosylase
*vapD*	333	VapD of *Haemophilus somnus*	73%/84	virulence-associated protein D
*ResA*	555	resolvase of *Azotobacter vinelandii* DJ	56%/183	resolvase
*MarR*	432	ESA_01699 of *Enterobacter sakazakii* ATCC BAA-894	28%/27	transcriptional regulator, MarR family
*Zeta-1*	1206	CAMGR0001_1552 of *Campylobacter gracilis* RM3268	50%/280	Zeta toxin
*Epsilon-1*	186			Epsilon antitoxin
*Zeta-2*	1209	Zeta toxin protein of *Verminephrobacter eiseniae* EF01-2	41%/296	Zeta toxin
*Epsilon-2*	255	Bcen2424_6818 of *Burkholderia cenocepacia*	30%/69	Epsilon antitoxin
*11375*	522	GCWU000324_02316 of *Kingella oralis* ATCC 51147	40%/77	Hypothetical
*tetM*	1935	TetM of *Streptococcus pneumoniae*	98%/644	tetracycline resistance
*11390*	474	Bcen2424_6818 of *Burkholderia cenocepacia*	29%/72	Epsilon antitoxin?
*11395*	897	YpIP275_pIP1202_0130 of *Yersinia pestis* biovar Orientalis	63%/297	DNA modification methylase
*11400*	342	NmucA2_00935 *Neisseria mucosa* ATCC 25996	36%/103	Hypothetical
*11405*	369	HMPREF0530_1319 *Lactobacillus paracasei*	36%/100	Hypothetical
*11410*	360	NgonSK11410 of *Neisseria gonorrhoeae* SK-92-679	100%/119	Hypothetical
*yegA*	558	YegA of *Neisseria gonorrhoeae* NCCP11945	40%/163	Conserved hypothetical with TAT signal sequence and DUF88 domain
*11420*	471	DR_0894 of *Deinococcus radiodurans* R1	28%/88	transcription elongation factor
*traC*	3000	TraC of IncP-1 plasmid pKJK5	46%/1032	DNA primase
*traD*	165	BACUNI_04470 of *Bacteroides uniformis* ATCC	56%/25	conjugal transfer
*traE*	2196	TraE of Birmingham IncP-alpha plasmid	58%/734	DNA topoisomerase III family
*traF*	513	TraF of *Bordetella pertussis*	40%/166	conjugal prepilin peptidase
*traG*	1890	TraG of *Bordetella pertussis*	69%/621	conjugal coupling protein
*traI*	2394	TraI of plasmid pB3	34%/809	DNA relaxase
*traJ*	378	TraJ of plasmid QKH54	45%/109	oriT-binding protein
*traK*	420	XfasM23_2251 of *Xylella fastidiosa*	30%/113	DNA transfer
*traL*	726	TraL of *Xylella fastidiosa* Dixon	48%/241	conjugal transfer
*traM*	483	TraM of *Legionella drancourtii* LLAP12	22%/110	DNA transfer
*kfrC*	537	KfrC of *Pseudomonas aeruginosa*	48%/148	plasmid partitioning
*kfrB*	357	TraO of *Bordetella pertussis*	41%/86	plasmid partitioning
*korB*	918	ParB of *Xylella fastidiosa* Dixon	48%/327	plasmid partitioning DNA binding protein
*incC*	756	IncC2 of Birmingham IncP-alpha plasmid	54%/257	plasmid partitioning ATP binding protein
*kleE*	246	KleE of Bordetella pertussis	28%/76	plasmid partitioning
*korC*	348	KorC of *Bordetella pertussis*	36%/75	plasmid partitioning, regulator
*trfA*	879	TrfA of *Nitrosomonas eutropha* C91	47%/265	plasmid replication, oriV activator
*ssb*	384	Ssb of *Xylella fastidiosa* M23	37%/115	single-strand DNA binding protein

### Analysis of the sequence of conjugative plasmids with the American *tetM* determinant

**Figure 2:**
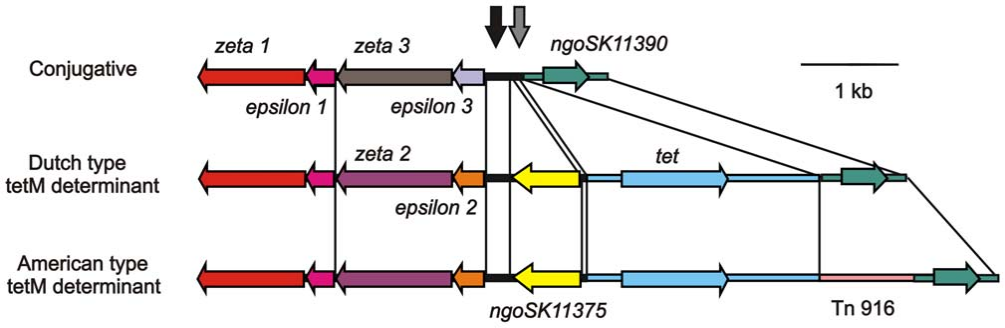
Comparison of the genetic loads regions of the conjugative plasmid, and the plasmids with the American and Dutch *tetM* determinants. The genetic load region between *zeta_1* and *ngoSK11390* is shown. Conserved regions are depicted in similar colors. Regions of 300 and 109 bps which are maintained after insertion of *ngoSK11375* are depicted by the black and grey arrows respectively.

Since the restriction patterns of the Dutch and American type conjugative plasmids showed some overlap [18], we tried to detect the presence of the different regions of the Dutch type plasmid in the isolates containing the American type *tetM* determinant. Region specific PCRs targeting the conjugative DNA-transfer (*traE*, *traD* and *traI*), mating-pair-formation (*trbB*, *trbC*, *trbD*, *trbE* and *trbI*), replication (*trfA* and *ssb*), stable plasmid inheritance and control (*korC*, *traN*, *traO*) regions and the regions used for gap closing of the initial sequences gave PCR fragments for all 6 isolates containing the American type tetracycline plasmid. Sequencing of the above regions revealed that these regions were 100% similar with the sequences determined for the plasmid with the Dutch *tetM* determinant. Isolation and restriction with *Bgl*I of the plasmids with the American type *tetM* determinant confirmed that these plasmids indeed contained the plasmid backbone of Dutch type conjugative with the American type *tetM* determinant integrated (data not shown). The region from the *zeta_1* gene to the n*gonSK11390* gene of a plasmid (pEP5050) with an American type *tetM* determinant and a Dutch type plasmid backbone isolated from clinical isolate 5050 was amplified and sequenced. Sequencing of this region revealed that the only difference between the two plasmids is the presence of a region with homology to the transposon Tn916 in the plasmid with the American type *tetM* determinant (see figure 2). This difference was reported previously for plasmids with the American type *tetM* determinant in an American type conjugative plasmid [16]. The *tetM* and the *ngonSK11390* gene show 96% and 99% identity with the genes present in the Dutch type plasmid, while the other genes were 100% identical (see Figure S1). Dutch type conjugative plasmids with an American type *tetM* determinant have been observed before at low frequency [33], but our results show that in our Dutch isolates, they occur with a very high frequency. Importantly, this shows that the *tetM* determinant is not a good marker to differentiate between conjugative plasmids with American and Dutch type backbones.

### Plasmids with *tetM* determinants are derived from the 24.5 MDa conjugative plasmid

Since it was proposed that the 25.2 MDa plasmid with a Dutch type backbone is a derivative of the 24.5 MDa conjugative plasmid by an insertion of the Dutch type *tetM* determinant [5], [21], it was tried to detect the presence of the different regions of the Dutch type plasmid described above in the isolates which were not positive for the presence of the Dutch and American *tetM* determinants. Remarkably, in 19/30 isolates the presence of the different regions could be detected, showing that 31/42 of our isolates contain a conjugative plasmid with a Dutch type backbone (see table 1). Again, sequencing of the above regions revealed that these regions were 100% similar with the sequences determined for the plasmid with the Dutch *tetM* determinant. This demonstrated that the conjugative plasmids with Dutch and American type *tetM* determinants are derived from the 24.5 MDa conjugative plasmid by insertion of the *tetM* determinant.

When the antibiotic resistance spectrum of the different clinical isolates was compared with the presence or absence of the conjugative plasmids, it was remarkable that all strains with resistance against the fluoroquinolones ciprofloxacin and oxacillin contained a conjugative plasmid. Resistance to fluoroquinolones is generally a result of point mutations in the DNA gyrase *gyrA* or the topoisomerase IV *parC* genes [34]. However, there is no clear indication in the plasmid sequence that would explain a correlation between presence of the conjugal plasmid and the resistance to fluoroquinolones.

To determine the exact position of the insertion of the *tetM* determinants in the conjugative plasmid, the region between the *zeta_1* and the *ngoSK11390* gene of plasmid pEP5233, a conjugative plasmid with a Dutch type backbone isolated from clinical isolate 5233 was amplified and sequenced. Alignment of the sequences (see Figure S1) showed that sequences outside the *epsilon_1* and the *ngoSK11390* genes are conserved between this plasmid (pEP5233) and the plasmids with the *tetM* determinant (pEP5289 and pEP5050) but that differences are found in the region between these genes (see figure 2). Conservation of 300 and 109 bp long regions in all three plasmids suggests that three insertions/rearrangements have taken place: I) insertion of the *tetM* determinant (with or without the Tn916 transposon), and II) insertion of the *ngoSK11375* gene and III) a rearrangement/mutagenesis of the *zeta_2*/*epsilon_2* genes to the *zeta_3/epsilon_3* genes. A more detailed analysis of the genetic load regions of these plasmids will be given below.

### The conjugative plasmids with the Dutch type backbone are phylogenetically divergent from the other IncP1 sub-families

**Figure 3:**
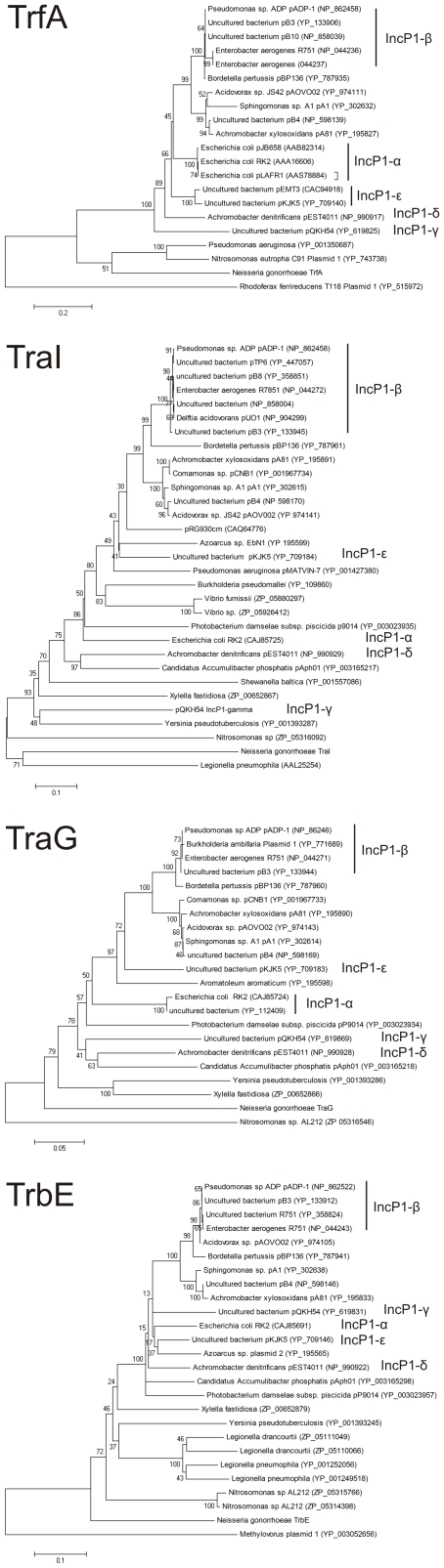
Phylogenetic analysis of genes of different IncP1 plasmids. The TraI (A), TrbE (B), TraG (C) and TrfA (D) proteins of the neisserial conjugative plasmids and of representative members of different IncP1 plasmid subfamilies (α, RP4; β, pADP-1; γ, QKH54; δ, pEST4011 and ε, pKJK5) representative for different regions of the conjugative plasmids were used to create phylogenetic trees of the IncP1 plasmid family. (For details see materials and methods).

The modular arrangement of the conjugative plasmid and derivates thereof, the absence of several genes often found in the IncP1-α subfamily, and an insertion of an additional module suggested that these plasmids are members of the IncP1-β sub-family. To verify placement of the plasmids in the IncP1-β sub-family, a phylogenetic analysis was performed on the TraI and TraG proteins encoded in the Tra region, the TrbE and TrbC proteins encoded in the Trb region, and the TrfA and KorB proteins encoded within the Rep and Ctl regions. Neighbor-joining tree containing representative members of the different IncP1 plasmid subfamilies (α, RP4; β, pADP-1; γ, QKH54; δ, pEST4011 and ε, pKJK5) and the neisserial conjugative plasmids with the Dutch type backbone were created (See figure 3). Remarkably this showed that these neisserial conjugative plasmids fall within the IncP1 family, but are phylogenetically divergent from the other IncP1 sub-families, and are members of a new subfamily.

### Analysis of the open reading frames of the IncP backbone

**Figure 4:**
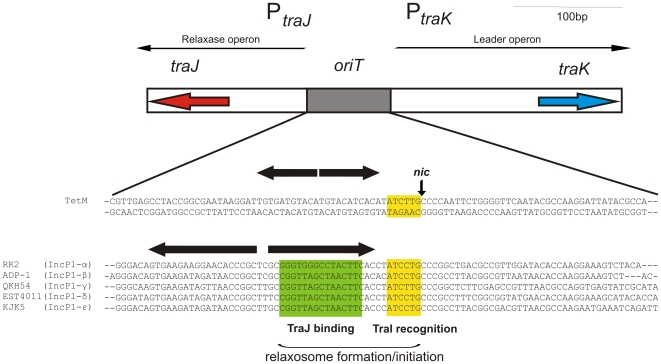
Comparison of the *oriT* sequences of different members of the IncP1 subfamilies. The common structure of the origin of transfer between the *traJ* and *traK* genes of the different IncP1 plasmids is shown. The *oriT* sequences of the indicted plasmids are depicted. In yellow and green the TraI recognition and TraJ binding sites are indicated. The large horizontal arrows indicate inverted repeat sequences adjacent to the *nic* site. The small vertical arrow indicates the (putative) *nic* sites of the relaxases. (The figure is modified from Pansgrau and Lanka (1996))

**Figure 5:**
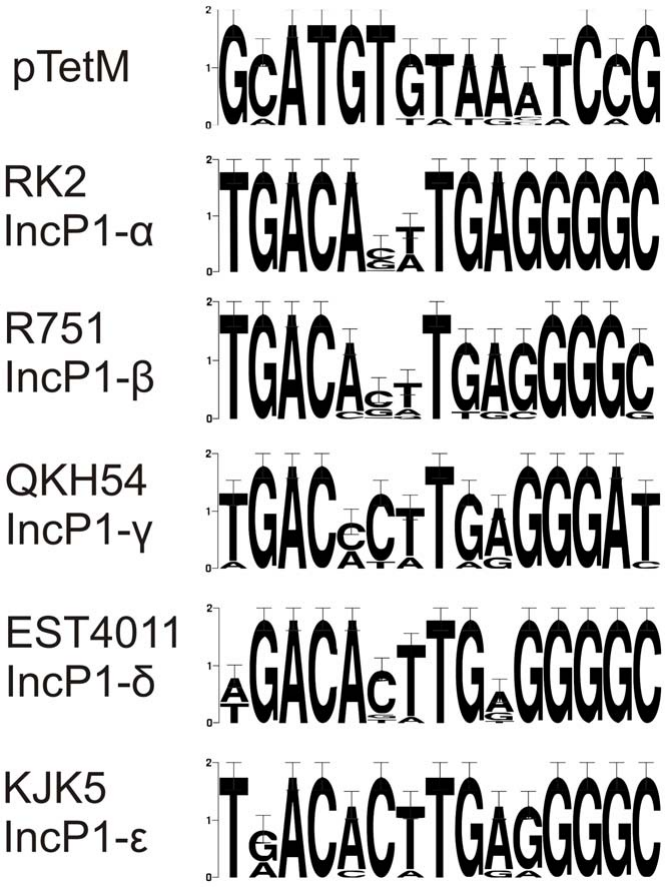
Comparison of the *oriV* iterons of different members of the IncP1 subfamilies. Weblogos were created to compare the 13 bp iterons of different members of the IncP1 subfamilies. The height of each base represents its conservation.

The conjugative DNA-transfer (Tra) region contains the transfer proteins, encoded in two oppositely transcribed *traCDEFGHIJ* and *traKLM* operons, and is similar to the other IncP-1 family plasmids. The origin of transfer (*oriT*), including the *nic* site is in plasmids of the IncP family located between these two operons [35]. This region also contains an inverted repeat sequence, which is the binding sequence for TraJ. The sequence in this region of the Neisserial conjugative plasmids differs significantly from the sequences observed in other IncP1 subfamilies. A putative *nic* site and an inverted repeat sequence are present, but the sequence of the inverted repeat to which TraJ putatively binds seems not to be conserved (See figure 4). The proteins encoded within the mating-pair-formation region, TrbA-TrbN, are similar to the proteins of the other IncP1-α, β, γ and ε plasmids. In pEST4011, a member of the IncP1-δ subfamily most of the Trb region (the C-terminal half of TrbE and the TrbF to TrbN proteins) is missing, but the TrbA-TrbD proteins are similar to the pTetM proteins. A difference with the other IncP1 subfamilies is found in TrbK. The *trbK* gene is the only non essential gene in the *trb* operon, and normally encodes a small lipoprotein involved in entry exclusion [36]. The protein located between TrbJ and TrbL in the Neisserial conjugative plasmids is a small protein with a signal sequence followed by a cysteine residue for lipid anchoring, but has very little homology in its mature part to the known TrbK proteins. However, the genetic location of the protein, the small size and the presence of the lipoprotein signal sequence suggests that this protein also is an entry exclusion protein, and we have therefore also named it TrbK. Similar to representatives of the IncP1-β and IncP1-γ subfamilies, the TrbO and TrbP proteins present in the IncP1-α and IncP1-ε families are not found in neisserial conjugative plasmids. The origin of replication (*oriV*) is in IncP1 plasmids generally located next to the replication initiation region. However in many plasmids insertions occur in this region, separating the *oriV* and the replication initiation region. Similar to the other IncP1 plasmids, the Rep region of the neisserial conjugative plasmids contains the *trfA* and *ssb* genes, which encode the *oriV* activator, and a single stranded DNA binding protein, respectively. No insertions have occurred between the Rep region and the *oriV*. The *oriV* region of IncP1 plasmids is approximately 400 bp long and consists of several direct repeats called iterons (17-mers) to which TrfA binds. Indeed the *trfA* gene is followed by 8 5′-GCATGTGTAAATCCG-3′ repeats in the next 400 bp. Comparison with the iterons of the other IncP1 subfamilies shows that the TrfA binding motif differs significantly from the other TrfA binding motifs (see figure 5). The inheritance and control region is relatively small compared to the other IncP1 plasmids, and contains only 6 genes. The IncC and KorB proteins are the ParA ATPase, and the ParB DNA binding protein homologues of the active partitioning system. KorB of the RK2 plasmid recognizes a 13 bp inverted repeat sequences (5′-TTTAGCCGCTAAA-3′), which was found 12 times on the RK2 plasmid [37]. This sequence is found 6 times on the neisserial conjugative plasmid, and 4 out of the 6 repeats were extended to 19 bp inverted repeats (5′-AATTTTAGCCGCTAAATAA-3′). Remarkably the putative ParB site located in the genetic load region, shows homology over 52 bp (with 86% identity) with the putative ParB site between KorC and KleE. Remarkably even though the genetic load region around this ParB site seems a spot for insertions of gene sequences, the ParB binding site in the genetic load region was conserved in all three conjugative plasmids, suggesting an important function for this region. The control region further contains the accessory stability components KfrB, KfrC, and KleE, and the KorC regulator. This region does not contain a post-segregational killing system, but a remarkable combination of such systems was found in the genetic load region.

### Analysis of the genetic load region

The genetic load region of the conjugative plasmid contains 14 open reading frames, putatively located in three larger operons, and two single genes (*ngonSK11390* and *vapD*). The plasmids with a *tetM* determinant contain next to the *tetM* determinant a second inserted region which contains the *ngonSK11375* gene. The *ngonSK11375* gene encodes a protein with an unknown function. The first putative operon consists of the *ngonSK11395*, *ngonSK11400* and *ngonSK11405* genes and contains the *ngonSK11395* gene which has homology to DNA modification methylases and two hypothetical proteins. This operon is separated from the operon including the *ngonSK11410*, *yegA* and *ngonSK11420* genes by a large 76 bp inverted repeat which functions putatively as a terminator. The *yegA* gene encodes a conserved protein with a Twin Arginine Translocation (TAT) signal sequence and a DUF88 domain, while the *ngonSK11410* and *ngonSK11420* genes encode hypothetical proteins. A homolog of the *yegA* gene is found within the Gonococcal Genetic Island, but also the function of this gene is unknown. The *zeta_1, epsilon_1, zeta_2* and *epsilon_2, marR* and *resA* genes also form a putative operon. In the conjugative plasmid the *zeta* and *epsilon* genes located at the positions of the *zeta_2* and *epsilon_3* genes in the *tetM* containing plasmids have obtained extended mutations without affecting the reading frame, and were named *zeta_3* and *epsilon_3*. The Zeta and Epsilon proteins encode components of a toxin-antitoxin system which was found in plasmid maintenance systems of Gram positive organisms which use a post segregation killing mechanism. Normally, plasmids encoding Zeta and Epsilon toxin-antitoxin proteins are part of a three-component system which also contains a regulator (*omega*). The *marR* gene also encodes a regulator of the MarR family, but this regulator comes from a different family then the *omega* regulators. The specific mechanism of the Zeta toxin is still unknown, but overexpression of the Zeta toxin of plasmid pSM19035 of *Streptococcus pyogenes* in *Bacillus subtilis* and *Escherichia coli* inhibited replication, transcription, and translation, and eventually lead to cell death [38]. This is the first Zeta/Epsilon toxin-antitoxin system identified in a Gram negative organism and the first Zeta/Epsilon toxin-antitoxin system in which two different copies of the toxin and of the antitoxin are located on one plasmid. Interestingly the genetic load region also contains the *vapD* gene that encodes the virulence associated protein D. VapD was shown to function as a toxin and is generally counteracted by an antitoxin protein called VapX. VapD/VapX systems have been reported in pathogens like *Rhodococcus equi* [39], *Dichelobacter nodosus* [40], *Xylella fastidiosa* [41], and *Haemophilus influenzae* [42]. It has been shown that expression of VapD enhances the invasion and survival within both human epithelial and endothelial cells [42]. Remarkably, no open reading frame encoding a VapX antitoxin could be identified within the sequence of the conjugative plasmid. A VapD/VapX toxin-antitoxin system was previously identified on pJD1, the cryptic plasmid which is present in 96% of the *N. gonorrhoea* strains [32]. It was tested whether the VapX antitoxin encoded on the cryptic plasmid pJD1 of *N. gonorrhoeae* might fulfill the role of antitoxin. Indeed when overnight conjugation experiments between *N. gonorrhoeae* containing the conjugative plasmid with the Dutch type *tetM* determinant and *Escherichia coli* were performed, tetracycline resistance was transferred much more efficiently to *E.coli* strains which expressed the VapX antitoxin of pJD1 (2 10^−2^ tetracycline resistant clones per recipient) then to *E.coli* strains that did not contain the antitoxin (1 10^−8^ tetracycline resistant clones per recipient). However, the conjugative plasmids could not be further maintained in *E.coli* for more then a few generations. The initial colonies did not grow in liquid medium, and could only be transferred once to new plates. This demonstrates that the conjugative plasmid can be conjugated to *E.coli*, but that the plasmid can not be further maintained. This suggests that the VapX antitoxin of the cryptic pJD1 plasmid might function as antitoxin of the VapD protein encoded on the conjugative plasmid, and might explain why the host range of the IncP1 conjugative plasmids of *N. gonorrhoeae,* is limited to *N. gonorrhoeae* and other neisserial species. This further suggests that the limitation of the host range could have resulted in a loss of the ability of the plasmid to be maintained in other species.

### Comparison with the type IV DNA secretion system encoded within the gonococcal genetic island

**Figure 6:**
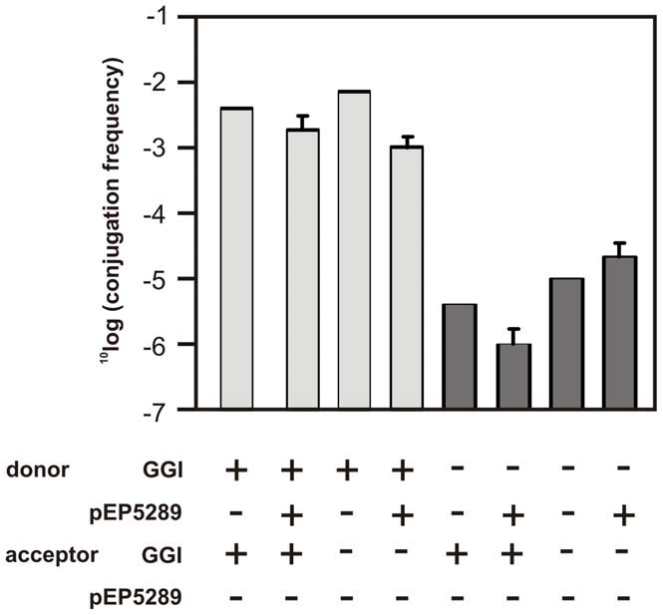
Transfer of chromosomal markers via the Type IV DNA secretion system is not influenced by the presence of the conjugative plasmid. Strains with the GGI (GGI+) or without the GGI (GGI-) and/or with (pEP5289+) or without (pEP5289-) the conjugative plasmid with the Dutch type *tetM* determinant and the Dutch type backbone were mixed, and grown at 37°C under 5% CO_2_. Donor strains contained the erythromycin marker in the *recA* gene, while recipient Neisseria strains contained the chloramphenicol marker. After 5 h of growth, serial dilutions were spread on selective media. Transfer frequencies were calculated as CFU of transconjugants per CFU of donor.

To examine whether there was a relation between the occurrence of the GGI and the conjugative plasmids, the presence of the GGI was determined for all strains. Within 27 of the 42 strains the presence of the GGI was detected. Conjugative plasmids were present in 14 of the 15 strains which do not contain the GGI and in 17 of 27 strains that contain the GGI. Thus the conjugative plasmids are present in strains which contain the GGI and in strains that do not contain the GGI. In a final step the involvement of the type IV DNA secretion systems encoded in the GGI and the conjugative plasmid in the spread of chromosomal markers was studied. Strain with and without the GGI, and with and without the conjugative plasmid were grown in the presence of acceptor strains with and without the GGI. In all donors strains the erythromycin marker was inserted within the *recA* gene to ensure one directional transfer, while the recipient strains contained the chloramphenicol marker. As was observed previously [4], the transfer of a chromosomal marker was increased 500-fold in donor strains that contained the type IV DNA secretion systems encoded in the GGI, whereas no influence was observed of the presence of the conjugative plasmids (see figure 6). This demonstrates that the type IV DNA secretion system encoded in the GGI is responsible for an increase in the spread of chromosomal markers, whereas no effects were observed for the conjugative plasmids.

## Discussion

Conjugative plasmids of *Neisseria gonorrhoeae* were found in a large percentage of clinical isolates obtained from the Public Health Laboratory in Amsterdam (The Netherlands). These plasmids all contained a Dutch type backbone. Remarkably, within the clinical isolates tested, no plasmids with an American type backbone could be detected. Within these plasmids with a Dutch type backbone insertions of both Dutch and American *tetM* determinants were found. Next to the insertion of the different *tetM* determinants, the only difference between the strains with the *tetM* determinant (25.2 MDa plasmids) and strains without the *tetM* determinant (24.5 MDa plasmids) was the insertion of the *ngoSK11375* gene. Previously several differences have been observed between the 25.2 and 24.5 MDa plasmids, e.g. 25.2 MDa plasmids showed a broader host range [17] and some plasmids could only be mobilized by a 25.2 MDa conjugative plasmid [29]. It is difficult to explain these differences based on the presence of the *tetM* determinant or the *ngoSK11375* gene, and it might be possible that these results have been obtained with 25.2 MDa plasmids with an American type plasmid backbone.

The conjugative plasmids with the Dutch type backbone are members of a novel IncP1 subfamily which similarly to other IncP1 plasmids contains backbone modules for replication initiation, conjugative DNA-transfer, mating-pair-formation, stable plasmid inheritance and control. The genes encoded in the backbone modules of the Dutch type neisserial conjugative plasmids are phylogentically divergent from the other IncP1 subfamilies, but since they belong to the broad host range IncP1 family, it is most likely that they have been aquired from other organisms. The limited host range compared to the normally broad host range of these plasmids might have allowed these plasmids to diverge from the other sub families.

The Dutch type neisserial conjugative plasmids contain a ‘genetic load’, module not found in other IncP1 plasmids. Remarkably three different putative toxin/antitoxin systems are located within this region. Two of the toxin/antitoxin systems belong to the Zeta/Epsilon toxin/antitoxin family. After insertion of the *tetM* determinant, several mutations occurred in one of the Zeta toxin/Epsilon antitoxin systems, generating mutated Zeta toxin and Epsilon antitoxin proteins. Zeta/Epsilon toxin/antitoxin systems have only been identified in Gram positive organisms [43], [44], [45] and always as a single copy. Although the structure of the Zeta/Epsilon toxin/antitoxin complex contained a phosphoryltransferase fold [46], the mechanism of the Zeta/Epsilon toxin/antitoxin system is still unknown. The functionality and the interactions between the different putative Zeta/Epsilon toxin/antitoxin systems encoded within the neisserial conjugative systems still need to be investigated.

The third toxin/antitoxin system identified on the neisserial conjugative plasmid belongs to the VapD family. The *vapD* gene has been previously identified in plasmids of *Rhodococcus equi* [39], *Actinobacillus actinomycetencomitans* [47], *Riemerella anatipestifer* [48] and *N. gonorrhoeae* [32], and on the chromosomes of *Haemophilus influenzae* [49] and *Xylella fastidiosa* [50]. In *Dichelobacter nodosus*, *vapD* was found both on the chromosome and on a plasmid [51]. The *vapD* gene has been associated with virulence in several of these facultative intracellular microorganisms [40], [42], [49], [52] but its precise role is still unknown. In *H influenzaea* the *vapX* gene was identified in the same operon as *vapD*. The VapX protein was shown to function as an antitoxin for VapD [42]. A homologue of the VapX protein was also identified next to the *vapD* gene on the cryptic pJD1 plasmid of *N. gonorrhoeae* [32]. Remarkably, the Dutch type conjugative plasmid of *N. gonorrhoeae* contains only the *vapD* gene. Since expression of the VapX antitoxin protein of pJD1 in *E.coli* acceptor cells strongly increased the transfer of the conjugative plasmid from *N. gonorrhoeae* to *E.coli*, we hypothise that VapX protein located on pJD1 might function as an antitoxin for VapD on the conjugative plasmid. This arrangement most likely prevent the conjugative plasmid to transfer to other cells that do not contain the cryptic pJD1 plasmid, limiting the host range of these conjugative plasmids to the host range of the pJD1 cryptic plasmid. Currently no information is available about a possible role of the VapD proteins encoded on the pJD1 and the conjugative plasmid on conjugation.

Multiple toxin/antitoxin systems have been found in many genomes, and some genomes contain more than 50 putative toxin/antitoxin systems [53]. Many different functions have been proposed for the multiple chromosomal toxin/antitoxin systems [54]. Most plasmids however contain only a single toxin/antitoxin system, and the advantages of having three different toxin/antitoxin systems on one conjugative plasmid also remains unclear.

## Materials and Methods

### Bacterial strains and growth conditions

The genomic DNA of clinical isolates and gonoccocal strains used in the study were obtained from the GG&GD Municipal Health Service, Public Health Laboratory in Amsterdam. The strains were characterized for their Maximal Inhibitory Concentrations for ciprofloxacin, tetracyclin and penicillin as described in [55]. *N. gonorrhoeae* was grown on GCB (Difco) plates containing Kellog's supplements [56] or in GCBL liquid medium (GCBL) containing 0.042% NaHCO_3_ [57] and Kellog's supplements. For *N. gonorrhoeae*, tetracycline was used at 5 µg/ml, chloramphenicol was used at 10 µg/ml and erythromycin at 10 µg/ml. For *E. coli*, tetracycline was used at 5 µg/ml, erythromycin was used at 500 µg/ml and ampicillin at 100 µg/ml. For further *N. gonorrhoeae* and *E. coli* strains used in this study see table 3.

**Table 3:** Strains used in this study.

strain	Genotype	source or references
*E.coli* TOP10F-	F- mcrA Δ(mrr-hsdRMS-mcrBC) ϕ80lacZΔM15 ΔlacX74 nupG recA1 araD139 Δ(ara-leu)7697 galE15 galK16 rpsL(Str^R^) endA1 λ^−^	Invitrogen
MS11A	*N.gonorrhoeae* strain	[62]
ND500	MS11AΔGGI	[4]
5289	low passage clinical isolate of *Neisseria gonorrhoeae*	GGD
5050	low passage clinical isolate of *Neisseria gonorrhoeae*	GGD
4393	low passage clinical isolate of *Neisseria gonorrhoeae*	GGD
4703	low passage clinical isolate of *Neisseria gonorrhoeae*	GGD
5371	low passage clinical isolate of *Neisseria gonorrhoeae*	GGD
5291	low passage clinical isolate of *Neisseria gonorrhoeae*	GGD
5233	low passage clinical isolate of *Neisseria gonorrhoeae*	GGD
EP006	MS11A ΔrecA::Erm	This study
EP030	ND500 ΔrecA::Erm	This study
EP015	MS11A lctP::Cm::aspC	This study
EP029	ND500 lctP::Cm::aspC	This study
EP006/5289	ΔrecA::Erm pTetM Dutch type	This study
EP030/5289	ΔrecA::Erm pTetM American type	This study

### Isolation of chromosomal DNA

Bacterial isolates were typically harvested for DNA isolation from pure culture plates, taking up to five morphologically identical and single colonies depending on the size of the colonies [55]. If no separate colonies grew on the pure culture plate, *N. gonorrhoeae* colonies were harvested from a tertiary plate. Colonies were lysed in 100 µl of 5 M guanidine thiocyanate buffer (BioMérieux, the Netherlands) containing 0.04 mg/ml glycogen (Roche Diagnostics, the Netherlands), and stored at 4°C until DNA isolation was performed. Chromosomal DNA was extracted from lysates by addition of an equal volume (700 µl) chilled (−20°C) isopropanol, followed by centrifugation for 20 minutes at 14.000 rpm. The pellet was subsequently washed twice with 500 µl 70% ethanol. Precipitated total DNA was dissolved in 50 µl 10 mM Tris/HCl (pH 8.0) and diluted 500 times in this buffer for most PCR reactions.

Alternatively, chromosomal DNA for PCR analysis was obtained by rapid cell lysis. Shortly, a smear of cells from overnight growth on GCB plates was resuspended in 25 µl of lysis solution (50 mM Tris/HCl pH 8.0, 20 mM EDTA, 50 mM NaCl). For each 25 µl PCR reaction 3 µl of the lysate was used. All sequencing reactions of PCR products were performed by Service XS (Leiden, the Netherlands).

### Determination of the *tetM* determinant and the presence of the Gonococcal Genetic Island

The presence of the *tetM* determinant was detected via PCR analysis as previously described. Shortly, either primers Tet_4F (universal forward) and Tet_AR (American reverse), or Tet_4F and Tet_DR (Dutch reverse) were used to amplify the *tetM* determinants, giving PCR fragments of 778 bp and 443 bp for the American and Dutch type determinants, respectively [31]. (For primers used in this study see table 4). Alternatively, primers RM4 and G1 were used to amplify the *tetM* determinant giving PCR fragments of 1600 and 700 bp for the American and Dutch type determinants, respectively [30]. The presence of the Gonococcal Genetic Island was detected via PCR analysis using the primers GGI-21F and GGI-22R (369 bp) and primers GGI-27F and GGI-28R (453 bp), amplifying regions of *traK* and *topB*, respectively. PCR products were analyzed by electrophoresis in 1% w/v agarose and visualized after ethidium bromide staining.

**Table 4:** Plasmids used in this study.

Plasmid	properties	source or references
pTrc99A	*E. coli* expression vector (AmpR)	[63]
pEP086	derivative of pTrc99A with mutated *trc* promotor	This study
pEP087	derivative of pEP086 containing *vapX*	This study
pIND1	*E. coli* insertion duplication mutagenesis vector for *N. gonorrhoeae*; contains *ermC*	[61]
pKH35	*E. coli* complementation vector for *N. gonorrhoeae* (CmR)	[4]
pEP013	derivative of pIND1 carrying fragment of *recA*	This study
pEP5050	*N. gonorrhoeae* conjugative plasmid with American type *tetM* determinant and Dutch type backbone isolated from clinical isolate 5050	This study
pEP5233	*N. gonorrhoeae* conjugative plasmid with Dutch type backbone isolated from clinical isolate 5233	This study
pEP5289	*N. gonorrhoeae* conjugative plasmid with Dutch type *tetM* determinant and Dutch type backbone isolated from clinical isolate 5289	This study

### Plasmid DNA isolation from *N. gonorrhoeae*

*Neisseria gonorrhoeae* clinical isolates 5050, 5233 and 5289 were chosen for isolation of their conjugative plasmids. The isolation was performed by a cleared lysate method followed by double acetate precipitation adapted from the Genome Sequencing Center, Washington University for *N. gonorrhoeae* as follows. Non-piliated colonies of *Neisseria gonorrhoea* were selected and transferred into 3 ml of GCBL medium GCBL liquid medium (GCBL) containing 0.042% NaHCO_3_ and Kellog's supplements with a final concentration of tetracycline (5 µg/ml) for plasmids containing the *tetM* determinant. After incubating for 3 hours at 37°C under 5% CO_2_ in a shaking incubator at 200 rpm, the culture was diluted to an OD_600_ of 0.2 in 6 tubes of 3 ml of GCBL containing 0.042% NaHCO_3_ and Kellog's supplements and growth was continued for 4 h. Then, all 18 ml of the pre-culture was added to 1 liter of GCBL containing 0.042% NaHCO_3_ and Kellog's supplements and incubated overnight at 37°C under 5% CO_2_ in a shaking incubator at 200 rpm. After harvesting the cells by centrifugation at 8.000 rpm in a JLA-16.25 rotor for 15 minutes, the cells were resuspended in 40 ml of 10 mM of EDTA, pH 8.0. After mixing gently, the solution was incubated at room temperature for 5 minutes. 80 ml of alkaline lysis solution (0.2 N NaOH and 1% SDS) was added and after gentle swirling until the solution became homogenous, it was incubated for 5 minutes at room temperature. Then, 60 ml cold, 3 M KOAc pH 5.5 was added and again mixed gently by swirling the bottle several times. Consecutively, the bottle was frozen at −20°C overnight. After thawing, the lysate was cleared from precipitated SDS, proteins, membranes, and chromosomal DNA by centrifugation at 10.000 rpm in a JLA-16.25 rotor for 15 minutes. Prior to re-centrifugation, the cleared supernatant was filtered to remove any floating material. Then, remaining insoluble material was removed by an additional centrifugation at 10.000 rpm in a JLA-16.25 rotor for 15 minutes. To the supernatant an equal volume of isopropanol was added and mixed by swirling. After centrifugation at 5.000 rpm in a JLA-16.25 rotor for 15 minutes, the supernatant was decanted and the pellet drained. The second acetate precipitation step was performed after gently dissolving the DNA pellet in 18 ml of 10∶50 TE, then 9 ml of 7.5 M KOAc (without pH adjustment) was added, and after mixing, the bottles were frozen and stored overnight at −80°C. After the solution was thawed and centrifuged at 6.000 rpm in a SS-34 rotor for 10 minutes. Dnase-free Rnase A was added to a final concentration of 100 ug/ml to the supernatant followed by incubation at 37°C for 1 hour. Then, 30 ml of cold 95% ethanol was added, and after mixing by inverting, the tubes were incubated at 4°C for 15 minutes. After centrifugation for 30 minutes at 3.000 rpm in a SS-34 rotor the pellet was washed with 30 ml of 70% ethanol and dried. Each pellet was resuspended in 1 ml of 10 mM Tris pH 8.0 and 0.1 mM EDTA and incubated at 37°C for 2 hours to ensure all the high molecular weight DNA was dissolved completely. The DNA was further stored at 4°C. Approximately 1.2 µg of plasmid DNA was obtained from 1 liter of *N. gonorrhoeae*. 15 ug of plasmid DNA obtained from 13 liters of *N. gonorrhoeae* clinical isolate 5289 was send to the Macrogen Corporation (Seoul, South Korea) to obtain the plasmid sequence via shotgun cloning and sequencing.

### Southern blot and restriction analysis

Whole plasmid DNA was transferred to nitrocelullose membranes by the method of Southern [58] followed by the hybridization of DIG-labelled *tetM*. The *tetM*-specific probe (448 bp) was PCR-amplified using primers Tet_4F and Tet_DR with purified plasmid as a template. The Dutch-type plasmid has been verified by the restriction analysis as follows. 10 µg of the purified Dutch type plasmid was digested for 14 h with *Bgl*I (0.1 U/µg) (Fermentas) followed by electrophoresis in 1% w/v agarose and visualized after ethidium bromide staining.

### DNA sequence analysis and annotations

The nucleotide sequence of the pEP5289 plasmid of *Neisseria gonorrhoeae* clinical isolate 5289 was determined via the shotgun method by Macrogen Inc. (Seoul), resulting in 7 contigs with at least 6 times coverage. Gap closure of the sequences was achieved by PCR amplification with the following primer combinations: trfA_F and ssb_R, trbB_F and trbC_R, korC_R and kleE_1F, 11390_F and 11395_R, trbN_F and res_R, res_F and zetaR. The analysis of predicted open reading frames was performed manually. The predicted open reading frames and their products were analyzed using the BLAST algorithm [59] and manually annotated. To determine the region in which the *tetM* determinant had putatively inserted into the 24.5 MDa conjugative plasmid (pEP5233) PCR reactions were performed with the following primers combinations: 11375_F and 11390_dw_R, 11375_F and 11390_R, Tet_up_1F and 11390_dw_R, Tet_up_1F_ and 11390_R, Tet_up_4F and 11390_dw_R, Tet_up_4F and 11390_R, Tet_up_6F and 11390_dw_R, Tet_up_6F and 11390_R. The resulting PCR products were send for sequencing either directly or after cloning in pGemT-Easy (Promega). To compare the plasmid core of the Dutch (pEP5289) and American (pEP5050) *tetM* type plasmids, with the 24.5 MDa conjugative plasmid (pEP5233) PCR reactions were performed with the primers used for the gap closure, and the additional PCR primer pairs: traO_R and traN_F for the *traN-traO* region, traI_R and traI_F for the *traI* gene, trbB_F and trbB_R for the *trbB* gene, trbI_F and trbI_R for the *trbI*, kleE_2F and trfA_R for the replication region, trbC_F and trbE_R for *trb* operon and traD_F and traE_R for *tra* operon. The templates for PCR reactions were the Dutch (pEP5289) and American (pEP5050) *tetM* type plasmids and the 24.5MDa conjugative plasmid (pEP5233). All PCR products were purified by gel elution (Sigma) and sequenced. The sequence of the conjugative plasmid with the Dutch type *tetM* determinant (pEP5289), and of the genetic load regions of the conjugative (pEP5233) and the plasmid with the American type *tetM* determinant (pEP5050) were deposited under genbank numbers GU479464, GU479465 and GU479466.

### Phylogenetic analysis

To generate a phylogenetic tree, homologues of the TraI, TrbE, TraG and TrfA proteins of representative members of the different IncP1 plasmid subfamilies (α, RP4; β, pADP-1; γ, QKH54; δ, pEST4011 and ε, pKJK5) and the Dutch type plasmids were obtained using BLAST [59] against the NCBI non redundant protein database using expect values lower then 1E-10 for TraI and TrfA, and expect values lower then 1E-100 for TraG and TrbE. Duplicates and incomplete proteins were removed, and multiple alignments were constructed with the CLUSTALX program [60] and an initial phylogenetic tree was created using the MEGA 4 package. Sequences of the IncP1 plasmid and of the Dutch type plasmids grouped in a small section of the phylogenetic tree. The sequences of this small section were selected, realigned with CLUSTALX, and Neighbor-joining trees based on the distance parameter were constructed using the MEGA 4 package. Bootstrap values from 1000 replicates were also acquired.

### Construction of strains and plasmids used in this study

Gonococcal strains were constructed as follows. Strains EP006 and EP030 were generated via the insertion-duplication method as described previously [61]. Briefly, an internal fragment of the *recA* gene was cloned in pIND1 plasmid [61] followed by transformation to strain MS11A and its derivative ND500 (MS11/ΔGGI), respectively. Conjugation of the tetracycline plasmid pTetM from non-piliated strain 5289 to piliated strains EP006 and EP030 resulted in piliated and tetracycline resistant strains EP006/5289 and EP030/5289, respectively. Strains EP015 and EP029 were constructed by transformation of strain MS11A and its derivative ND500 (ΔGGI) with plasmid pKH35 [4]. VapX was cloned using introduced *Nco*I and *Xba*I sites behind the mutated *trc* promoter of pEP086 after PCR amplification of the *vapX* gene of the pJD1 plasmid contained in strain 5289 using primers 503 (vapX_F) and 504 (vapX_R). pEP086 is derived from vector pTrc99A. In pEP086, expression was reduced after mutagenesis of the promotor by PCR amplification of plasmid pTrc99A with primers 475 (pTrc99A mut-10_F) and 476 (pTrc99A mut-10_R). Plasmids used and created in this study are listed in table 5.

**Table 5:** Primers used in this study.

Oligo	Oligo name	
234	Tet_4F	5′-CTCGAACAAGAGGAAAGC-3′
235	Tet_AR	5′-GCATTCCACTTCCCAAC-3′
236	Tet_DR	5′-TGCAGCAGAGGGAGG-3′
298	Tet_1R	5′-CCACTGTTATATAATAAGCTTTCTGTTAAGG-3′
299	Tet_7F	5′-TTGCCAGCCCCGTCGTCCAAATAGTCGGAT-3′
304	Tet_up_3F	5′-ATAAACTGTCAATTTGATAGTGGGAAC-3′
305	Tet_dw_1R	5′-CCATATTTATATAACAACATAAAATACACTAAGTT-3′
306	Tet_8R	5′-ACCTTCTGTTTGATTACAAT-3′
307	Tet_9F	5′-TACAGTCATTTATATGGAGAGACCGT-3′
309	Tet_3F	5′-GGAAAATGGGGATTCCCACAATCT-3′
310	Tet_6R	5′-TACAATCCTTGTTCACAGCCAT-3′
324	Tet_2R	5′-TGTCCTGGCGTGTCTATGAT-3′
393	trbC_R	5′-GTAACGGACTGGCGGAGATT-3′
394	trbN_F	5′-CTTGCCGGTAGGCAAGGGAA-3′
395	ssb_R	5′-AGGCCAAGAACGCCGTCAGG-3′
396	trbB_F	5′-AGAGGAACAGGGAGGCAATT-3′
397	korC_R	5′-ATGCCTATTGGGTTTTATTG-3′
398	trfA_F	5′-TAATCTACAAGCTGCATCCA-3′
399	kleE_1F	5′-CCCATTACGATACCGAACGTCAAG-3′
400	11395_R	5′-GTTACATGGAAAGCCGCCGTAAGAC-3′
401	traO_R	5′-AGCAGAAAAACAAAGAGTGCTGG-3′
402	traN_F	5′-ACGGTTCAGCCTTGATTGTG-3′
403	traI_R	5′-TATCATCTGATGGTATCCCTTGCG-3′
404	traI_F	5′-ACCGATCTGATTTCATCCTT-3′
405	trbB_F	5′-AGCGTGATTTGGGAAATGAG-3′
406	trbB_R	5′-GGCATTGTTTGCATGCAGTG-3′
407	trbI_F	5′-TGCACTGGTGGCAGCAACTA-3′
408	trbI_R	5′-TGGTTGTAAACGCGCTCCAC-3′
409	Tet_10R	5′-CCATAACTGCATTTTGAAAC-3′
410	11390_F	5′-CGCCCGAATCTTGCTGTATT-3′
412	Zeta_F	5′-TTCATCCCCTTGGCTTCAGC-3′
414	Res_R	5′-AAGAGCAGAATGTGGCCGCA-3′
415	11375_F	5′-CTTGGACGGTTTTCATGTTC-3′
416	Tet_up_1F	5′-CCAGTTGTCTTTGTTCAGTC-3′
417	Tet_up_4F	5′-GGAACAAATAATTAGATGTCCT-3′
418	Tet_up_6F	5′-CAGTTTAAGAATACCTTTATCATGTGATTC-3′
419	11390_R	5′-AGCCGTCTGCGAGATGTAGG-3′
420	11390_dw_R	5′-ACACCCCCTGTCCAAGCGAAATTT-3′
421	Tet_up_5F	5′-TTTTTAGGAGGGCTTAGTTTTTTGTACC-3′
422	Res_F	5′-GATCTTCATACCACCGTTCG-3′
423	Zeta_R	5′-GGGCAACACTGCAACGCAAA-3′
433	Zeta_F	5′-CCAGCATTTCTACCCCGTAC-3′
434	Tet_up_2R	5′-AGTGCGGCTGGATACGCCGTTAG-3′
435	RM4	5′-CCAAATCCTTTCTGGGCT-3′
436	G1	5′-ATCACTCACAGTTAATTTT-3′
474	11355_F	5′-ATGACATCATCGAACTTGTCGATAAAT-3′
475	pTrc99a mut -10 F	5′- TTCCTTGTGTGGAATTGTGAGCGGATAACAATTTC – 3′
476	pTrc99a mut -10 R	5′- CGAGCCGGATGATTAATTGTCAACA – 3′
477	kleE_2F	5′-CCTCCGTCCGATATAGTTTG-3′
478	trfA_R	5′-GCCAGCCGTATTGAATACAG-3′
79	trbC_F	5′-CGCACAGCATTACCTTCTTC-3′
480	trbE_R	5′-GGCCGTTCAATTCCATAACC-3′
481	traD_F	5′-TCTGCGATCACTTCGATGTC-3′
482	traE_R	5′-TGTGCGATCCGGAAGATTAC-3′
503	vapX_F	5′- TGCGCCATGGAAACCACTATCCCCACCGGTTC – 3′
504	vapX_R	5′- GCGCTCTAGATTAGGCAGCCTTTGGGTCTTGAACG – 3′
GGI-21F	traK_F	5′- GGGGGTACCACGGAACTTGAGCAGAATCG- 3′
GGI-22R	traK_R	5′- AAAGGATCCGTAGTGCCACGCATCATAGA- 3′
GGI-27F	topB_F	5′- CGCGGATCCCTGGCCTTGGTCGGAATAAT- 3′
GGI-28R	topB_R	5′- AAAGGTACCCTGGCGTAATACTGACGGAT- 3′
148	recA_F	5′- GAGCTCCGTGTGCGCCTTTGTCGATG – 3′
149	recA_R	5′- GGTACCGTTTCGTCCTGCGTCCCTTC – 3′

### Conjugation to Escherichia coli

Non-piliated gonococcocal donor strain 5289 was streaked from O/N grown plates and suspended in 3 ml of GCBL (Difco) medium supplemented with Kellogg's supplements and 0.042% NaHCO_3_. The culture was diluted till an OD_600_ of 0.2 and was grown with shaking for 4 h at 37°C in a 5% CO_2_ atmosphere_._ The recipient strain *Escherichia coli* TOP10 was diluted 50-fold from an overnight culture and was grown for 3 h. Equal portions of donor and recipient were mixed, centrifuged and pellets were resuspended in 100 µl of GCBL. The suspensions were placed on GCB plates in the presence or absence of 0.5 mM IPTG. Plates were incubated at 37°C under 5% CO_2_ overnight. After the incubation cells were suspended in 100 µl of LB medium, and mating was interrupted by vigorous vortexing for 5 s and serial dilutions were plated on selective media (5 µg/ml of tetracyline for transconjugants selection). The mating efficiency was calculated as a CFU of transconjugants per CFU of recipient.

### Neisseria gonorrhoeae conjugation assay

Donors and recipients from plates inoculated overnight were suspended in 3 ml of GCBL (Difco) medium supplemented with Kellogg's supplements and 0.042% NaHCO_3_. Cultures were diluted till an OD_600_ of 0.2 and were grown for 4 h at 37°C with 5% CO_2._ Equal portions of donor and recipient were mixed, centrifuged and pellets were resuspended in 100 µl of GCBL. The suspensions were placed on GCB plates supplemented with Kellogg's supplements. Plates were incubated at 37°C under 5% CO_2_ for 24 hours. After the incubation cells were suspended in 1 ml of GCBL (Difco) medium supplemented with Kellogg's supplements and 0.042% NaHCO_3_ [57] and serial dilutions were plated on selective media. Recipient *Neisseria gonorrhoeae* strains contained the chloramphenicol marker while donors were tetracycline resistance. All donors had the erythromycin insertion within the *recA* gene to ensure one directional transfer. Transfer frequencies were calculated as CFU of transconjugants per CFU of donor.

### Transformation assay

Donors and recipients from plates inoculated overnight were suspended in 3 ml of GCBL (Difco) medium supplemented with Kellogg's supplements and 0.042% NaHCO_3_. Cultures were grown for 2.5 h at 37°C with 5% CO_2._ 1 ml of both donor and recipient cultures were centrifuged and pellets were resuspended in 3 ml of GCBL. 0.5 ml of donor and 0.5 ml of recipient were inoculated in 3 ml of GCBL for 5 h at 37°C with 5% CO_2_ (both shaking and non shaking conditions). Serial dilutions were spread on selective media. Recipient *Neisseria* strains contained the chloramphenicol marker while donors were tetracycline resistance. All donors had the erythromycin marker inserted within the *recA* gene to ensure one directional transfer. Transfer frequencies were calculated as CFU of transconjugants per CFU of donor.

### Additional information

Weblogos were created at http://weblogo.berkeley.edu/.

## Supporting Information

Figure S1 Alignment of the region between the zeta_1 and ngoSK11390 of the genetic load region of the neisserial conjugative plasmids. Depicted are the conjugative plasmid with the American type tetM determinant (A), the Dutch tetM determinant (D) and the plasmid without a tetM determinant (C). The different genes are indicated with different colors. Arrows above the genes indicate the orientation of the genes.
(0.23 MB DOC)
