# Supplementary material for: Conjugative Plasmids of Neisseria gonorrhoeae
Source: PLoS One. 2010 Apr 1;5(4):e9962. doi: 10.1371/journal.pone.0009962 (PMC2848598; doi:10.1371/journal.pone.0009962)
Supplement: Figure S1 — Alignment of the region between the zeta_1 and ngoSK11390 of the genetic load region of the neisserial conjugative plasmids. Depicted are the conjugative plasmid with the American type tetM determinant (A), the Dutch tetM determinant (D) and the plasmid without a tetM determinant (C). The different genes are indicated with different colors. Arrows above the genes indicate the orientation of the genes. (0.23 MB DOC) [file pone.0009962.s001.doc]

Pachulec and van der Does Supplementary figure 1

**A** TAATCAAGCGAAATTTCCGTGATCAACCGCATTTTTTCTTTCATTTCCGTATCCCTGCTT

**D** TAATCAAGCGAAATTTCCGTGATCAACCGCATTTTTTCTTTCATTTCCGTATCCCTGCTT 1

**C** TAATCAAGCGAAATTTCCGTGATCAACCGCATTTTTTCTTTCATTTCCGTATCCCTGCTT

**ZETA 1**

**A** ATCTGCTGATGGATTTTTTGGCGGAATACTCCAAAGACTTGACAATCAATTTGCCCTTAT

**D** ATCTGCTGATGGATTTTTTGGCGGAATACTCCAAAGACTTGACAATCAATTTGCCCTTAT 2

**C** ATCTGCTGATGGATTTTTTGGCGGAATACTCCAAAGACTTGACAATCAATTTGCCCTTAT

**ZETA 1**

**A** TGCTTCCGATAGAAACGTTCTGCCCTATCTCAACCAATTTGGCCAGTCTTTCTTCGTTAT

**D** TGCTTCCGATAGAAACGTTCTGCCCTATCTCAACCAATTTGGCCAGTCTTTCTTCGTTAT 3

**C** TGCTTCCGATAGAAACGTTCTGCCCTATCTCAACCAATTTGGCCAGTCTTTCTTCGTTAT

**ZETA 1**

**A** GTGTTGCATTGTGCCTGACAAGCCCGTTTTTGGTTTTCTGAAATACCGCAGCGGCATCCT

**D** GTGTTGCATTGTGCCTGACAAGCCCGTTTTTGGTTTTTTGAAATACCGCAGCGGCATCCT 4

**C** GTGTTGCATTGTGCCTGACAAGCCCGTTTTTGGTTTTTTGAAATACCGCAGCGGCATCCT

**ZETA 1**

**A** TGTAAATGATTTCTCCGGAATAAGTTTGACCAGTCTCTGCTTTTTTAGCTTGACCTAGTA

**D** TGTAAATGATTTCTCCGGAATAAGTTTGACCAGTCTCTGCTTTTTTAGCTTGACCTAGTA 5

**C** TGTAAATGATTTCTCCGGAATAAGTTTGACCAGTCTCTGCTTTTTTAGCTTGACCTAGTA

**ZETA 1**

**A** AATAGATACCGGCTTTCATGCCTGTTACAGGAATTTGTTCCCCCTTAGACCACAACTCGT

**D** AATAGATACCGGCTTTCATGCCTGTTACAGGAATTTGTTCCACCTTAGACCACAACTCGT 6

**C** AATAGATACCGGCTTTCATGCCTGTTACAGGAATTTGTTCCACCTTAGACCACAACTCGT

**ZETA 1**

**A** TTGCTGACTTTGTTTCTAGGGTAACACCCTTGGAGTTAGTAACTACCCTTTGCTTGGAAT

**D** TTGCTGACTTTGTTTCTAGGGTAACACCCTTGGAGTTAGTAACTACCCTTTGCTGGGAAT 7

**C** TTGCTGACTTTGTTTCTAGGGTAACACCCTTGGAGTTAGTAACTACCCTTTGCTGGGAAT

**ZETA 1**

**A** ACTCTTTTTTGGCGAAAATACTTTCAAGATCCATACTGCCCTTGGCCTGATTGAAACATT

**D** ACTCTTTTTTGGCGAAAACACTTTCAAGATCCATACTGCCCTTGGCCTGATTGAAGCATT 8

**C** ACTCTTTTTTGGCGAAAACACTTTCAAGATCCATACTGCCCTTGGCCTGATTGAAGCATT

**ZETA 1**

**A** CCCGGCTGATTTTCCCTGAATCGAAATCACCTTGGATTTTCTCGGCCAACCTGCCCAATA

**D** CCCGGCTGATTTTTCCTGAGTCGAAGTCGCTCTGGATTTTCTCGGCCAACCTGCCCAATA 9

**C** CCCGGCTGATTTTTCCTGAGTCGAAGTCGCTCTGGATTTTCTCGGCCAACCTGCCCAATA

**ZETA 1**

**A** TTTGTTCTTGCGACCCCAAGGAAAGCATTTTAATGACATCATCGAATTTATCGATAAATT

**D** TTTGCTCTTGCGACCCCAAGGAAAGCATTTTGATGACATCATCGAACTTGTCGATAAATT 10

**C** TTTGCTCTTGCGACCCCAAGGAAAGCATTTTGATGACATCATCGAACTTGTCGATAAATT

**ZETA 1**

**A** CACTGTTCCTGTCTTGGTTGATGCCGACTATTTTTACCGCATCGCCAAACTTATCCCTGA

**D** CACTGTTCCTGTCTTGGTTGATACCGACAATTTTTACCGCATCACCAAACTTATCCCTGA 11

**C** CACTGTTCCTGTCTTGGTTGATACCGACAATTTTTACCGCATCACCAAACTTATCCCTGA

**ZETA 1**

**A** TTTGACTGAGCCCGTTAGGAAGATTAGCCTGAATATCGGCCATGATGCCTATACTAGCTC

**D** TTTGCTTGAGTCCGTCAGGAAGATTGGCCTGAATATCAGCCATAATACCTATACTGGCTC 12

**C** TTTGCTTGAGTCCGTCAGGAAGATTGGCCTGAATATCAGCCATAATACCTATACTGGCTC

**ZETA 1**

**A** CCCTACCATATTCGTTAAACCGTTTGTAAGTATTGTCGGAAGCTCTCTCAGCCCTCATGC

**D** CCCTGCCGTATTCGTTAAACCGTTTGTAAGTATTGTCGGAAGCCCTTTCAGCCCTCATGC 13

**C** CCCTGCCGTATTCGTTAAACCGTTTGTAAGTATTGTCGGAAGCCCTTTCAGCCCTCATGC

**ZETA 1**

**A** TGACGGCCACAATAATAACTTCGAGGCCACGATCCAAACATTGCTCAATCTTGCGAAAAG

**D** TGACGGCTACGATAGTAACTTCAAGATTACGCTCCAGACACTGCTCAATCTTGCGAAAAG 14

**C** TGACGGCTACGATAGGAACTTCAAGATTACGCTCCAGACACTGCTCAATCTTGCGAAAAG

**ZETA 1**

**A** CAGACTGCGGCCGCGCCAGCTGTCCTTCAAAAAGCAGCTTGGTGGTATCCTGCATCATCA

**D** CAGACTGCGGCCGTGCAAGCTGTCCTTCAAAAAGCAGCTTGGTGGTATCCTGCATCATCA 15

**C** CAGACTGCGGCCGTGCAAGCTGTCCTTCAAAAAGCAGCTTGGTGGTATCCTGCATCATCA

**ZETA 1**

**A** TGGTCTTTACTGTCGAAGTTTTTCCCGAACCCGGAATTCCCGTAACGAAAATAACTTCTT

**D** TGTTTTTTACTGTCGAAGTTTTTCCCGAACCCGGAATTCCCGTAACGAAAATAACTTCTT 16

**C** TGTTTTTTACTGTCGAAGTTTTTCCCGAACCCGGAATTCCCGTAACGAAAATAACTTCTT

**ZETA 1**

**A** TCTTCTGTGGCTCGTCGCGTTTCAGCACCTCGTCAAACTGGGTAGAAGATAAAACTGCTG

**D** TCTTCTGTGGCTCGTCGCGTTTCAGTACCTCGTCAAACTGGGTAGAAGATAAAACTGCTG 17

**C** TCTTCTGTGGCTCGTCGCGTTTCAGTACCTCGTCAAACTGGGTAGAAGATAAAACTGCTG

**ZETA 1**

**A** CTGAATTGTGGATAGCATTATTAACCGTTGCCCTGTCTTCTTCGTTTTCAAAAGCTGGAA

**D** CTGAATTGTGGATGGCATTATTAACCGTTGCCCTGTCTTCTTTGTTCTCAAAAGCTGGAA 18

**C** CTGAATTGTGGATGGCATTATTAACCGTTGCCCTGTCTTCTTTGTTCTCAAAAGCTGGAA

**ZETA 1**

**A** ACAACTCTTTAAACGTATCAGCACAAACATATCTACCGCCTTCTGTATCTGCATGTTGGC

**D** ACAACTCTTTAAACGTATCAGCACAAACATATCTACCGCCTTCTGTATCTGCATGTTGGC 19

**C** ACAACTCTTTAAACGTATCAGCACAAACATATCTACCGCCTTCTGTATCTGCATGTTGGC

**ZETA 1**

**A** TATATAAAGACAATATCTCGTCTGTTTGTTCGGTCGCAAAACGGATGGCCTCATCCTGAA

**D** TATATAAAGACAATATCTCGTCTGTTTGTTCGGTCGCAAAACGGATGGCCTCATCCTGAA 20

**C** TATATAAAGACAATATCTCGTCTGTTTGTTCGGTCGCAAAACGGATGGCCTCATCCTGAA

**ZETA 1**

**A** CGGAAGACAAATATTCGTTATTGCCAAAATCTCTCAAATTAATATCGCTAGACAGTTTTA

**D** CGGAAGACAAATATTCGTTATTGCCAAAATCTCTCAAATTAATATCGCTAGACAGTTTTA 21

**C** CGGAAGACAAATATTCGTTATTGCCAAAATCTCTCAGATTAATATCGCTAGACAGTTTTA

**EPSILON 1**

**ZETA 1**

**A** CCATAGCGGATTATTGCTCCTTATTTGCCGCCCACAACACTCTCAACTTAGCTAGAAGCT

**D** CCATAGCGGATTATTGCTCCTTATTTGCCGCCCACAACACTCTCAACTTAGCTAGAAGCT 22

**C** CCATAACGGATTATTGCTCCTTATTTGCCGCCCACAACACTCTTAACTTAGCTAGAAGCT

**EPSILON 1**

**A** CCGGAGATGGGCCGGCATACAGGCGTTTTTCCACTTCTTTGCGGAAGGCTTCGTCTGTCA

**D** CCGGAGATGGGCCGGCATACAAGCGTTTTTCCACTTCTTTGCGGAAGGCTTCGTCTGTCA 22

**C** CAGGAGATGGGCCGGCATACAGGCGTTTTTCCACTTCTTTGCGGAAGGCTTCGTCTGTCA

**EPSILON 1**

**A** TCATCCTGCGGTTTTTTTCGCAAGCCTCTTTTATCATTCTGATAGCATTACTTTCTTGGG

**D** TCATCCTGCGGTTTTTTTCGCAAGCCTCTTTTATCATTCTGATAGCATTACTTTCTTGGG 23

**C** TCATCCTGCGGTTTTTTTCGCAAGCCTCTTTTATCATTTTGATAGCATTACTTTCTTGGG

**EPSILON 1**

**ZETA 2**

**A** GCTCAACTTTATTCATAATAAAATCCTAATCTTAATTTTATATTACTCATCATCGCTTCA

**D** GCTCAACTTTATTCATAATAAAATCCTAATCTTAATTTTATATTACTCATCATCGCTTCA 24

**C** GCTCAACTTTATTCATAATAAAATCCTAATCTTAATTTTATATTACTCATTATCGCTTCA

**ZETA 2**

**A** TCGTTTTCTTTAATGAGGCATCTGCCGGTTTAACGGACAATTCATTTCCTTCCCGCTTAA

**D** TCGTTTTCTTTAATGAGGCATCTGCCGGTTTAACGGACAATTCATTTCCTTCCCGCTTAA 25

**C** TCGTTTTCTTTACGGAAGCGTCCGATGCTTTGACGGACAACCCATTTCCTTCCCGCTTGA

**ZETA 2**

**A** TAGAAACCTTCTGACCAATTTCCACCTGTTCGGACAAACTAGAAAACTTTCCTTCCGCCA

**D** TAGAAACCTTCTGACCAATTTCCACCTGTTCGGACAAACTAGAAAACTTTCCTTCCGCCA 26

**C** TGGAAACTTTCTGACCAATTTCCACCTGTTCGGACAGACTGGAGAATTTCCCCTCCGACA

**ZETA 2**

**A** TACCCTGAACCGCCTTATGGCGTATCAGACCTTGGTCAGTCTTCTGGAAAACCGATGCCT

**D** TACCCTGAACCGCCTTATGGCGTATCAGACCTTGGTCAGTCTTCTGGAAAACCGATGCCT 27

**C** TTCCCTGAACCGCCTTATGGCGTATCAGACCTTGGTCTGTCTTCTGAAAAACTGATGCCT

**ZETA 2**

**A** CGTCTTTATGGACAATCTCT-CCGCTGTATTCCCTGCCGG-ATTTAGCCGGTTTCGCC-G

**D** CGTCTTTATGGACAATCTCT-CCGCTGTATTCCCTGCCGG-ATTTAGCCGGTTTCGCC-G 28

**C** CGTCTTTATGGACAATCTCTTCCACTGTACTCTTTGCCGGGATTCGGCCGGTTTCGCCCG

**ZETA 2**

**A** TGCCAAGCATGTGAATACCGGCTTTCATCCCCTTGGCTTCAGCCTTCTCAATATCCTGCC

**D** TGCCAAGCATGTGAATACCGGCTTTCATCCCCTTGGCTTCAGCCTTCTCAATATCCTGCC 29

**C** TGCCAAGCATGTGAATACCGGCTTTCATCCCCTTGGCTTCAGCCTTCTCAATATCCTGCC

**ZETA 2**

**A** ATGTGTCGCCTGATTTGCGTTGCAGTGTTGCCCCTTCTGAATTAGCCACCACCCTTTCCT

**D** ATGTGTCGCCTGATTTGCGTTGCAGTGTTGCCCCTTCTGAATTAGCCACCACCCTTTCCT 30

**C** ATGTGTCGCCTGATTTGCGTTGCAGTGTTGCCCCTTCTGAATTGGCCACCACCCTTTCCT

**ZETA 2**

**A** GACGGTAAATCTCTTCCACACGCTGATAAATTGCTTCACTATGCTGTTTGACCATGCCGA

**D** GACGGTAAATCTCTTCCACACGCTGATAAATTGCTTCACTATGCTGTTTGACCATGCCGA 31

**C** GACGATAAATCTCTTCCACACGCTGATAGATCTCTTCCCGGTGTTGCTTTACCTTGTCAA

**ZETA 2**

**A** GATAATTAGGTATTGGATCGATACTGCGGCTTTCTGCCTGAAGCTGTATCTCGTCCCATG

**D** GATAATTAGGTATTGGATCGATACTGCGGCTTTCTGCCTGAAGCTGTATCTCGTCCCATG 32

**C** AATAATTTGGAACAGGATCTTGGGAACGCCTGTCTGCCTGAAGCTGTATCACATCCCAGA

**ZETA 2**

**A** CTTTTACAACCTGTTTCAACCGTTTAGGGGTTATTTCCTGATAAGCCGATAGTGCATCTT

**D** CTTTTACAACCTGTTTCAACCGTTTAGGGGTTATTTCCTGATAAGCCGATAGTGCATCTT 33

**C** CTTGAGCAACCTGCTTCAAACGTTCAGGAGTAATCTGCTGATAAAACTCCATAGCATCTT

**ZETA 2**

**A** TTGCCGTTTCATACTGGTTTTGTCGCCGATTCAAACTGTCATAAAGGATGTCTCCTGCGC

**D** TTGCCGTTTCATGCTGGTTTTGTCGCCGATTCAAACTGTCATAAAGGATGTCTCCTGCGC 34

**C** TGGCACTCGCCTGTTTGTTCTGTTGTTTATTCAGGCTGTCATACAGGATTTCCCCGGGGC

**ZETA 2**

**A** GGTTGGTAACACGAACCCTGTCAAAACTTGATTCACATTGAGACAACGTATCCTTGAATC

**D** GGTTGGTAACACGAACCCTGTCAAAACTTGATTCACATTGAGACAACGTATCCTTGAATC 35

**C** GATTGGTTACGCGGATGCGGTCGAATTCCGCCTCATGAGTTGCAACTGTATTTTTAAATC

**ZETA 2**

**A** CAGCCATTGCCTCGTTATGGTACGACTCTTCAACAAATCTAGGCTGTGAAACACCTTTGG

**D** CAGCCATTGCCTCGTTATGGTACGACTCTTCAACAAATCTAGGCTGTGAAACACCTTTGG 36

**C** CCTCAAACGCCTTATTGTGAAAATCTTCGTCAACGAAACGCGGTTGGATATTCTTAGTAA

**ZETA 2**

**A** CATGTTGTTCCTCATAACGCTTAAAAATCCCGGCTACACTCTCTTCAGAAGCAGTGGCTA

**D** CATGTTGTTCCTCATAACGCTTAAAAATCCCGGCTACACTCTCTTCAGAAGCAGTGGCTA 37

**C** GATACTGATCTTCATACCGTTTAAAAATACCGGCCAAACTTTCTTCCGGAGCAGTCGCAA

**ZETA 2**

**A** CCGCCAGCATTTCTACCCCGTACCCTTCTGATTTTCTATCACGGATAAATTGGCTGATAC

**D** CCGCCAGCATTTCTACCCCGTACCCTTCTGATTTTCTATCACGGATAAATTGGCTGATAC 38

**C** CGGCCAACATTTCGATTTTCAGTCCCGCTTCTCGCGCGGCTTTAATGCTCATGCCTACTG

**ZETA 2**

**A** TTGCAGCATTTCTAAATGTCCCCTCCTCCACAATATTCCGCCGGTTTTCTTTTGCACTAT

**D** TTGCAGCATTTCTAAATGTCCCCTCCTCCACAATATTCCGCCGGTTTTCTTTTGCACTAT 39

**C** CTTCAGCATTACGGAAAGTTCCTTCTTCCAAAACGTTCCGGGAGTTTTCCAATGCCGATT

**ZETA 2**

**A** TCCGAACACTAATGGCCAAAGCACCGGCATCTTTCTGAGTTTGTTCAGACGAATACACCA

**D** TCCGAACACTAATGGCCAAAGCACCGGCATCTTTCTGAGTTTGTTCAGACGAATACACCA 40

**C** TTCGTACTCCTACAGCCAACTTGCCTGCATCTTCTTGTGTCTGCTGAGACGAATAAACGA

**ZETA 2**

**A** CCCCCTCCGGCGCAGGAATCAGTGCACGCATAATATCTGCATCCACATGGATATATCCGC

**D** CCCCCTCCGGCGCAGGAATCAGTGCACGCATAATATCTGCATCCACATGGATATATCCGC 41

**C** CTCCAGGAGGAACTGGTATTTTTTGCCGCATAATGTCGGCATCAACATGTATATGCCCTC

**ZETA 2**

**A** CTTCTTGGCGTAACTCGCTTTTAGCTAAAGAAGCTGCCTTACTCTTACCCGCTCCGGGTT

**D** CTTCTTGGCGTAACTCGCTTTTAGCTAAAGAAGCTGCCTTACTCTTACCCGCTCCGGGTT 42

**C** CCTTTGAAGCGAACTCTTCTTTTACGATGTCGGCGGTCTTACTTTTTCCCGCTCCCGGTT

**ZETA 2**

**A** GTGCCCCAACTAGAACAATTTTGGGTTGCTCCACACTTTGCGTTCTAGATAAACGTGGCT

**D** GTGCCCCAACTAGAACAATTTTGGGTTGCTCCACACTTTGCGTTCTAGATAAACGTGGCT 43

**C** GAGCCGCTACCAAGGCGATCTGCGGCACATCTACCGCCTGTGTAATTTCCAAAAGAGCAG

**ZETA 2**

**A** TCATGAAACCATTCAGAATTCTTGCCTTACCTTCGGCACTTAAAGGGGTGTACTCAATCA

**D** TCATGAAACCATTCAGAATTCTTGCCTTACCTTCGGCACTTAAAGGGGTGTACTCAATCA 44

**C** ACATATATTCGGCAACAATTTCGGCGCGTCCCGCGTCGCTTAACTTTTGGTAATCACTCA

**EPSILON 2**

**A** TTTTACACGTCCAACTGTTTGGTCATCCGGATTGAAAATACGCGCGATAATATCTGTGTC

**D** TTTTACACGTCCAACTGTTTGGTCATCCGGATTGAAAATACGCGCGATAATATCTGTGTC 45

**C** TACAGAACCCCCAACAATCGTATCATTGGGATCCAAAATCCTTGCGATGACATCTTCGTC

**EPSILON 2**

**A** CTCCGGAGAGAGGCTGTCACGCAACATATCGATAGCCTCAAGCCTTGCTGTACAGTATTC

**D** CTCCGGAGAGAGACTGTCACGCAACATATCGATAGCCTCAAGCCTTGCTGTACAGTATTC 46

**C** CGTTGCTCTTAAGTTATGGCGGAATACTGTAATCGCTTTAATACTATCCGAACAATATTG

**EPSILON 2**

**A** GATAAACGCTTGGTTTGGTTTATCCTTTACAAACTCGGACTCTCTTGCCTGAATTAATGG

**D** GATAAACGCTTGGTTTGGTTTATCCTTTACAAACTCGGACTCTCTTGCCTGAATTAATGG 47

**C** AATGAATGCTTCATTTGGATGAGATTTTTTCCGCTCCTCTTCTCTAGCCTGAATAAACGG

**EPSILON 2**

**A** GCGCATCGAATCTCCCAACAACTCCAAAGCCACTTCATAATTGATACCAGGAATAGTTTC

**D** GCGCATCGAATCTCCCAACAACTCCAAAGCCACTTCATAATTGATACCAGGAATAGTTTC 48

**C** TTGGGCGGCATTACCCAAAACCTCTAACGCTACTTCGTAATTGATACCCGGAATTACTTC

**EPSILON 2**

**A** TTGCATTTGCATCTTGGCTTCCTCCAAAAGTTATCCACAAATTTTGTTAGTAAGCTGCCC

**D** TTGCATTTGCATCTTGGCTTCCTCCAAAAGTTATCCACAAATTTTGTTAGTAAGCTGCCC 49

**C** TTTCATTT----TTTGGTTCCCTCCAAAAATTATCCACAAATTTTGTTAGTAAGCTGCCC

**A** ATATCGCCAGCCTGCCACATATAATAAATTGAATCTTCGTCATCAAACCGTTTTTGCCTG

**D** ATATCGCCAGCCTGCCACATATAATAAATTGAATCTTCGTCATCAAACCGTTTTTGCCTG 50

**C** ATATCGCCAGCCTGCCACA**C**ATAATAAATTGAATCTTCGTCATCAAACCGTTTTTGCCTG

**A** CTTTTTAAGCAACATGGTTCAAAACCTATGTTTGAATAATAAGCCAGTTCAAAATAAATA

**D** CTTTTTAAGCAACATGGTTCAAAACCTATGTTTGAATAATAAGCCAGTTCAAAATAAATA 51

**C** CTTTTTAAGCAACATGGTTCAAAACCTATGTTTGAATAATAAGCCAGTTCAAAATAAATA

**A** ACAACGTTTTTGACCTATACTTCCAGACCTTAACGAAACATGGTTCATAAAGTACACTTT

**D** ACAACGTTTTTGACCTATACTTCCAGACCTTAACGAAACATGGTTCATAAAGTACACTTT 52

**C** ACAACGTTTTTGACCTATACTTCCAGACCTTAACGAAACATAGTTCATAAAGTACACTTT

**A** TTGCACCTACTTATCTAAGTTACCCACCTCTTAATCAAGATTTGGCCATTGCCCACCATA

**D** TTGCACCTACTTATCTAAGTTACCCACCTCTTAATCAAGATTTGGCCATTGCCCACCATA 53

**C** TTGCACCCACTTATCTAGGTTACTCACCTCTTACTCAAGATTTGGCCATTGCCCACCATA

**A** TCCCATAATGTGTAGAAACCAGTAGCGTTGGGTGGATGACGGCGTTTCGGGTTTTCCGCA

**D** TCCCATAATGTGTAGAAACCAGTAGCGTTGGGTGGATGACGGCGTTTCGGGTTTTCCGCA 54

**C** CCCCATAATGGCTTTAATTGACTCCTGTCAAGCCCAAAGACTTCTTTCATGTTT------

**11375**

**A** GGCGGCAGGCAGCCTGCAATGGTAGTGGCTAATCCTGCGCGCTATTGCATGAGACACAAA

**D** GGCGGCAGGCAGCCTGCAATGGTAGTGGCTAATCCTGCGCGCTATTGCATGAGACACAAA 55

**C** ------------------------------------------------------------

**11375**

**A** CTTCAAGATAGCGCAGCAAGGCTTTTTCTATCGTTTGTCGATTAACATCAAGGTAGAAGA

**D** CTTCAAGATAGCGCAGCAAGGCTTTTTCTATCGTTTGTCGATTAACATCAAGGTAGAAGA 56

**C** ------------------------------------------------------------

**11375**

**A** GAGACTTATATTCGGATGCAATGCCTTTTGCTAGGTTGGCAATAAACATCTTGTCGTCTT

**D** GAGACTTATATTCGGATGCAATGCCTTTTGCTAGGTTGGCAATAAACATCTTGTCGTCTT 57

**C** ------------------------------------------------------------

**11375**

**A** CATCTATATCAATATGGCTGATAAAAGTCGGTTTGGCCAAATTCTTGACAGTAAGATAGG

**D** CATCTATATCAATATGGCTGATAAAAGTCGGTTTGGCCAAATTCTTGACAGTAAGATAGG 58

**C** -------------------------------------------------------------

**11375**

**A** CATTGAATTCTCCAACATTCATATCCGAGAAATAGTATTGCAGCTCTATCGGTTTCATTT

**D** CATTGAATTCTCCAACATTCATATCCGAGAAATAGTATTGCAGCTCTATCGGTTTCATTT 59

**C** ------------------------------------------------------------

**11375**

**A** TTTCTTCGATTTTGGCTTCAATATTCTTCACCGATTCCCGTCGTTGTGCTTCTTTCAGGC

**D** TTTCTTCGATTTTGGCTTCAATATTCTTCACCGATTCCCGTCGTTGTGCTTCTTTCAGGC 60

**C** -----------------------------------------------------------

**11375**

**A** AGCCTGAAAAGTTGTTTTTCAGTGCCAATGCGTGCAGCAGAGCGTACAGGGCATCGTTAC

**D** AGCCTGAAAAGTTGTTTTTCAGTGCCAATGCGTGCAGCAGAGCGTACAGGGCATCGTTAC 61

**C** -----------------------------------------------------------

**11375**

**A** CGGCTGCCGACAGCGGAGGCACGGATTCGAGCAGTCCGCCGGAGACGGTTTCGGCTGCCG

**D** CGGCTGCCGACAGCGGAGGCACGGATTCGAGCAGTCCGCCGGAGACGGTTTCGGCTGCCG 62

**C** -----------------------------------------------------------

**11375**

**A** CACCGTAGCGGCCGTTCTCTTTCGTCAGGCGGATAATGATTTCGTATTCTTGGACGGTTT

**D** CACCGTAGCGGCCGTTCTCTTTCGTCAGGCGGATAATGATTTCGTATTCTTGGACGGTTT 63

**C** ------------------------------------------------------------

**11375**

**A** TCATGTTCATACTCCCAGTTGTCTTTGTTCAGTCCGTGCTAACGGCGTATCCAGCCGCAC

**D** TCATGTTCATACTCCCAGTTGTCTTTGTTCAGTCCGTGCTAACGGCGTATCCAGCCGCAC 64

**C** ------------------------------------------------------------

**A** TCCGGCGGCGGCATCCTGCCCGACTCTCTTTCACGTTTATAATTATAGGATAATTTATCC

**D** TCCGGCGGCGGCATCCTGCCCGACTCTCTTTCACGTTTATAATTATAGGATAATTTATCC 65

**C** --------------------------------------ATAATTATAGGATAATTTATCC

**A** TACTTGTCAAATAAATTTTAGCCGCTAAATTTTTACTCTCGCTTATTTATTCATTATAAT

**D** TACTTGTCAAATAAATTTTAGCCGCTAAATTTTTACTCTCGCTTATTTATTCATTATAAT 66

**C** TACTTGTCAAATAAATTTTAGCCGCTAAATTTTTACTCTCGCTTATTTATTCATTATAAT

**A** AAATTAATCGTTATTTTGTTTAACTGTAAAATTGGAGATTCCTTGACAAATATGCTCTTA

**D** AAATTAATCGTTATTTTGTTTAACTGTAAAATTGGAGATTCCTTGACAAATATGCTCTTA 67

**C** AAATTAATTGTTATTCTGCTTAACTGCTA-------------------------------

**A** CGTGCTATTATTTAAGTGACTATTTAAAAGGAGTTAATAAATATGCGGCAAGGTATTCTT

**D** CGTGCTATTATTTAAGTGACTATTTAAAAGGAGTTAATAAATATGCGGCAAGGTATTCTT 68

**C** ------------------------------------------------------------

**A** AAATAAACTGTCAATTTGATAGTGGGAACAAATAATTGGATGTCCTTTTTTAGGAGGGCT

**D** AAATAAACTGTCAATTTGATAGCGGGAACAAATAATTAGATGTCCTTTTTTAGGAGGGCT 69

**C** -----------------------------------------------------------

**A** TAGTTTTTTGTACCCAGTTTAAGAATACCTTTATCATGTGATTCTAAAGTATCCAGAGAA

**D** TAGTTTTTTGTACCCAGTTTAAGAATACCTTTATCATGTGATTCTAAAGTATCCAGAGAA 70

**C** ------------------------------------------------------------

**A** TATCTGTATGCTTTGTATACCTATGGTTATGCATAAAAATCCCAGTGATAAAAGTATTTA

**D** TATCTGTATGCTTTGTATACCTATGGTTATGCATAAAAATCCCAGTGATAAAAGTATTTA 71

**C** ------------------------------------------------------------

**tetM**

**A** TCACTGGGATTTTTATGCCCTTTTGGGTTTTTGAATGGAGGAAAATCACATGAAAATTAA

**D** TCACTGGGATTTTTATGCCCTTTTGGGTTTTTGAATGGAGGAAAATCACATGAAAATTAA 72

**C** ------------------------------------------------------------

**tetM**

**A** TATTGGAGTTTTAGCTCATGTTGATGCAGGAAAAACTACCTTAACAGAAAGCTTATTATT

**D** TATTGGAGTTTTAGCTCATGTTGATGCAGGAAAAACTACCTTAACAGAAAGCTTATTATT 73

**C** ------------------------------------------------------------

**tetM**

**A** ATATAACAGTGGAGCGATTACAGAATTAGGAAGCGTGGACAAAGGTACAACGAGGACGGA

**D** ATATAACAGTGGAGCGATTACAGAATTAGGAAGCGTGGACAAAGGTACAACGAGGACGGA 74

**C** ------------------------------------------------------------

**tetM**

**A** TAATACGCTTTTAGAACGTCAGAGAGGAATTACAATTCAGACAGGAATAACCTCTTTTCA

**D** TAATACGCTTTTAGAACGTCAGAGAGGAATTACAATTCAGACAGGAATAACCTCTTTTCA 75

**C** ------------------------------------------------------------

**tetM**

**A** GTGGGAAAATACGAAGGTGAACATCATAGACACGCCAGGACATATGGATTTCTTAGCAGA

**D** GTGGGAAAATACGAAGGTGAACATCATAGACACGCCAGGACATATGGATTTCTTAGCAGA 76

**C** ------------------------------------------------------------

**tetM**

**A** AGTATATCGTTCATTATCAGTTTTAGATGGGGCAATTCTACTGATTTCTGCAAAAGATGG

**D** AGTATATCGTTCATTATCAGTTTTAGATGGGGCAATTCTACTGATTTCTGCAAAAGATGG 77

**C** ------------------------------------------------------------

**tetM**

**tetM**

**tetM**

**A** CGTACAAGCACAAACTCGTATATTATTTCATGCACTTAGGAAAATGGGGATTCCCACAAT

**D** CGTACAAGCACAAACTCGTATATTATTTCATGCACTTAGGAAAATGGGGATTCCCACAAT 78

**C** ------------------------------------------------------------

**tetM**

**A** CTTTTTTATCAATAAGATTGACCAAAATGGAATTGATTTATCAACGGTTTATCAGGATAT

**D** CTTTTTTATCAATAAGATTGACCAAAATGGAATTGATTTATCAACGGTTTATCAGGATAT 79

**C** ------------------------------------------------------------

**A** TAAAGAGAAACTTTCTGCCGAAATTGTAATCAAACAGAAGGTAGAACTGTATCCTAATAT

**D** TAAAGAGAAACTTTCTGCCGAAATTGTAATCAAACAGAAGGTAGAACTGTATCCTAATAT 80

**C** ------------------------------------------------------------

**A** GTGTGTGACGAACTTTACCGAATCTGAACAATGGGATACGGTAATAGAGGGAAACGATGA

**D** GTGTGTGACGAACTTTACCGAATCTGAACAATGGGATATGGTAATAGAAGGAAATGATTA 81

**C** ------------------------------------------------------------

**tetM**

**A** CCTTTTAGAGAAATATATGTCCGGTAAATCATTAGAAGCATTGGAACTCGAACAAGAGGA

**D** CCTTTTGGAGAAATATACGTCTGGGAAATTATTGGAAGCATTAGAACTCGAACAAGAGGA 82

**C** ------------------------------------------------------------

**tetM**

**A** AAGCATAAGATTTCAGAATTGTTCTCTGTTCCCTCTTTATCACGGAAGTGCAAAAAGTAA

**D** AAGCATAAGATTTCATAATTGTTCCCTGTTCCCTGTTTATCACGGAAGTGCAAAAAACAA 83

**C** ------------------------------------------------------------

**tetM**

**A** TATAGGGATTGATAACCTTATAGAAGTTATTACTAATAAATTTTATTCATCAACACATCG

**D** TATAGGGATTGATAACCTTATAGAAGTGATTACGAATAAATTTTATTCATCAACACATCG 84

**C** ------------------------------------------------------------

**tetM**

**A** AGGTCCGTCTGAACTTTGCGGAAAAGTTTTCAAAATTGAGTATTCGGAAAAAAGACAGCG

**D** AGGTCAGTCTGAACTTTGCGGAAAAGTTTTCAAAATTGAGTATTCGGAAAAAAGACAGCG 85

**C** ------------------------------------------------------------

**tetM**

**A** TCTTGCATATATACGTCTTTATAGTGGCGTACTGCATTTGCGAGATTCGGTTAGAATATC

**D** TCTTGCATATATACGTCTTTATAGTGGCGTACTGCATTTGCGAGATCCGGTTAGAATATC 86

**C** ------------------------------------------------------------

**tetM**

**A** GGAAAAGGAAAAAATAAAAATTACAGAAATGTATACTTCAATAAATGGTGAATTATGTAA

**D** GGAAAAGGAAAAAATAAAAATTACAGAAATGTATACTTCAATAAATGGTGAATTATGTAA 87

**C** ------------------------------------------------------------

**A** AATTGATAAGGCTTATTCCGGGGAAATTGTTATTTTGCAGAATGAGTTTTTGAAGTTAAA

**tetM**

**D** AATCGATAAGGCTTATTCCGGGGAAATTGTTATTTTGCAGAATGAGTTTTTGAAGTTAAA 88

**C** ------------------------------------------------------------

**tetM**

**A** TAGTGTTCTTGGAGATACAAAGCTATTGCCACAGAGAAAAAGAATTGAAAATCCGCACCC

**D** TAGTGTTCTTGGAGATACAAAGCTATTGCCACAGAGAGAGAGAATTGAAAATCCCCTCCC 89

**C** ------------------------------------------------------------

**tetM**

**A** TCTACTACAAACAACTGTTGAACCGAGTAAACCTGAACAGAGAGAAATGTTGCTTGGAAA

**D** TCTGCTGCAAACGACTGTTGAACCGAGCAAACCTCAACAAAGGGAAATGTTACTTGATGC 90

**C** ------------------------------------------------------------

**tetM**

**A** TCTCAGATAGTGATCCGCTTCTACGATATTACGTGGATTCTACG-----------ACACA

**D** ACTTTTAGAAATCTCCGACAGTGACCCGCTTCTGCGATATTATGTGGATTCTGCGACACA 91

**C** ------------------------------------------------------------

**tetM**

**A** TGAAATTATACTTTCTTTCTTAGGGAAAGTACAAATGGAAGTGATTAGTGCACTGTTGCA

**D** TGAAATCATACTTTCTTTCTTAGGGAAAGTACAAATGGAAGTGACTTGTGCTCTGCTGCA 92

**C** ------------------------------------------------------------

**tetM**

**A** AGAAAAGTATCATGTGGAGATAGAACTAAAAGAGCCTACAGTCATTTATATGGAGAGACC

**D** AGAAAAGTATCATGTGGAGATAGAAATAAAAGAGCCTACAGTCATTTATATGGAAAGACC 93

**C** ------------------------------------------------------------

**tetM**

**A** GTTAAAAAATGCAGAATATACCATTCACATCGAAGTGCCGCCAAATCCTTTCTGGGCTTC

**D** GTTAAAAAAAGCAGAGTATACCATTCACATCGAAGTTCCACCGAATCCTTTCTGGGCTTC 94

**C** ------------------------------------------------------------

**tetM**

**A** CATTGGTTTATCTGTATCACCGCTTCCGTTGGGAAGTGGAATGCAGTATGAGAGCTCGGT

**D** CATTGGTCTATCTGTAGCACCGCTTCCATTAGGGAGCGGAGTACAGTATGAGAGCTCGGT 95

**C** ------------------------------------------------------------

**tetM**

**A** TTCTCTTGGATACTTAAATCAATCATTTCAAAATGCAGTTATGGAAGGGATACGCTATGG

**D** TTCTCTTGGATACTTAAATCAATCGTTTCAAAATGCAGTTATGGAGGGGATACGCTATGG 96

**C** ------------------------------------------------------------

**tetM**

**A** TTGCGAACAAGGATTATATGGTTGGAATGTGACGGACTGTAAAATCTGTTTTAAGTATGG

**D** CTGTGAACAAGGATTGTATGGTTGGAATGTGACGGACTGTAAAATCTGTTTTAAGTATGG 97

**C** ------------------------------------------------------------

**tetM**

**A** CTTATACTATAGCCCTGTTAGTACCCCAGCAGATTTTCGGATGCTTGCTCCTATTGTATT

**D** CTTATACTATAGCCCTGTTAGTACCCCAGCAGATTTTCGGATGCTTGCTCCTATTGTATT 98

**C** ------------------------------------------------------------

**tetM**

**A** GGAACAAGTCTTAAAAAAAGCTGGAACAGAATTGTTAGAGCCATATCTTAGTTTTAAAAT

**D** GGAACAAGTCTTAAAAAAAGCTGGAACAGAATTGTTAGAGCCATATCTTAGTTTTAAAAT 99

**C** ------------------------------------------------------------

**tetM**

**A** TTATGCGCCACAGGAATATCTTTCACGAGCATACACCGATGCTCCTAAATATTGTGCGAA

**D** TTATGCGCCACAGGAATATCTTTCACGAGCATACAACGATGCTCCTAAATATTGTGCGAA 100

**C** ------------------------------------------------------------

**tetM**

**A** CATCGTAGACACTCAATTGAAAAATAATGAGGTCATTCTTAGTGGAGAAATCCCTGCTCG

**D** CATCGTAGACACTCAATTGAAAAATAATGAGGTCATTCTTAGTGGAGAAATCCCTGCTCG 101

**C** ------------------------------------------------------------

**tetM**

**A** GTGTATTCAAGAATATCGTAGTGATTTAACTTTCTTTACAAATGGACGTAGTGTTTGTTT

**D** GTGTATTCAAAAATATCGTAGTGATTTAACTTTCTTTACAAATGGACGTAGTGTTTGTTT 102

**C** ------------------------------------------------------------

**tetM**

**A** AACAGAGTTAAAAGGGTACCATGTTACTACCGGTGAACCTGTTTGCCAGCCCCGTCGTCC

**D** AACAGAGTTAAAAGGGTACCATGTTACTACCGGTGAACCTGTTTGCCAGCCCCGTCGTCC 103

**C** ------------------------------------------------------------

**tetM**

**A** AAATAGTCGGATAGATAAAGTACGATATATGTTCAATAAAATAACTTAGTGTATTTTATG

**D** AAATAGTCGGATAGATAAAGTACGATATATGTTCAATAAAATAACTTAGTGTATTTTATG 104

**C** ------------------------------------------------------------

**A** TTGTTATATAAATATGGTTTCTTGTTAAATAAGATGAAATATTTTTTAATAAAGATTTGA

**D** TTGTTATATAAATATGGTTTCTTGTTAAATAAGATGAAATATTTTTTAATAAAAATT--- 105

**C** ------------------------------------------------------------

**A** ATTAAAGTGTAAAGGAGGAGATAGTTATTATAAACTACAAGTGGATATTGTGTCCTGTTA

**D** ------------------------------------------------------------ 106

**C** ------------------------------------------------------------

**A** TGTGGAAATAAAACACGATTAAAGATAAGGGAAGATACTGAATTAAAAAAATTCCCCCTC

**D** ------------------------------------------------------------ 107

**C** ------------------------------------------------------------

**A** TATTGTCCGAAATGCAGACAAGAAAATTTAATTGAAATAAAGCAGTTCAAAGTAACTGTG

**D** ------------------------------------------------------------ 108

**C** ------------------------------------------------------------

**A** ATTACAGAGCCAGACGCAAAGACGCAGAGCCGATAAAATGAGATTAATACAATCTCATTT

**D** ------------------------------------------------------------ 109

**C** ------------------------------------------------------------

**Tn916**

**A** TATCGGCTCTTTCCGTTATGTATGGATTCTTTTAATTAGTCTTCGATGTTTCTTGCTTCG

**D** ------------------------------------------------------------ 110

**C** ------------------------------------------------------------

**Tn916**

**A** TTGATACCGCTGGCTAAAGATTCCATTAAGGATAGGTCTTTGTCTGTAAAGCTATCCAAG

**D** ------------------------------------------------------------ 111

**C** ------------------------------------------------------------

**Tn916**

**A** TATTTCTCTATCGGTAATCGTCGGGTGCTTTTTACCAAGTTATTAGCAGGTAAGAAAAAT

**D** ------------------------------------------------------------ 112

**C** ------------------------------------------------------------

**Tn916**

**A** TCATCAACGGAAACATGAAGTAACGATACAAGGTCATAAAGAACTTGTATGCTGGGGTGT

**D** ------------------------------------------------------------ 113

**C** ------------------------------------------------------------

**Tn916**

**A** TGCCCTTTATTTTCAATATTAGTTAAGTACCGTGGGTCAATTTCAATCAATGCTCCCACT

**D** ------------------------------------------------------------ 114

**C** ------------------------------------------------------------

**Tn916**

**A** TGTTCACGAGTTAAACCTCGTTTCAATCGAGCTTCTTTAATGGCTAAACCAAAGGCTCTA

**D** ------------------------------------------------------------ 115

**C** ------------------------------------------------------------

**Tn916**

**A** AAATCATATTTATCTTCTTTTTTACGCATAGTAGACCACCTCTATACATTTTATTGTTCC

**D** ------------------------------------------------------------ 116

**C** ------------------------------------------------------------

**A** TACTGAATTAAAAACAGGTATAGAAAAACGTGTTATATGGTTTATAGGTTTATATTTAAT

**D** ------------------------------------------------------------ 117

**C** ------------------------------------------------------------

**A** AAAAAGCACTACTAAACGCCAATAAAAAAAACCGTTATATGGTAGTGCTATTTACGCTGT

**D** ------------------------------------------------------------ 118

**C** ------------------------------------------------------------

**A** TAAAATATTGTATATTACTTCCAAATGGCGGTTTGTTGGAGGTCAACGTCGCCATGAAGT

**D** ------------------------------------------------------------ 119

**C** ------------------------------------------------------------

**A** ACATCATATACAATAAATTTCCTTACATTGGGTTATCAAAAAAATTAACTGTGAGTAATT

**D** ----------------------------------------------AACTGTGAGTGATT 120

**C** ----------------------------------------AAAATTAACTGTGAGTGATT

**11390**

**A** GGAGACACCATGCCACATGAGCAAATGATTGATGCTTTTTCAGCAGTCATTCAAAACTCC

**D** GGAGACACCATGCCACATGAGCAAATGATTGATGCTTTTTCAGCAGTCATTCAAAACTCC 121

**C** GGAGACACCATGCCACATGAGCAAATGATTGATGCTTTTTCAGCAATCATTCAAAACTCC

**11390**

**A** TTAAGCCTATATGAAGAGCAAACTCCGATATACTACTACCTGACTATCTGCGGTTCTTGG

**D** TTAAGCCTATATGAAGAGCAAACTCCGATATACTACTACCTGACTATCTGCGGTTCTTGG 122

**C** TTAAGCCTATATGAAGAGCAAACTCCGCTATACTACTACCTGACTATCTGCGGTTCTTGG

**11390**

**A** GTTCTGACTGCACTCGGCATCCTCTCCTTGGTGTCCGTATTCTTTACCATCAAGCAGAAA 123

**D** GTTCTGACTGCACTCGGCATCCTCTCCTTGGTGTCCGTATTCTTTACCATCAAGCAGAAA

**C** GTTCTGACTGCACTCGGCATCCTCTCCTTGGCGTCCGTATTCTTTACCATCAAGCAGAAA

**11390**

**A** AAACCATACCGTTTTATCGTCCCGATTCTGTTTTTATGGCTGGGATATGAAAGCACCGAA 124

**D** AAACCATACCGTTTTATCGTCCCGATTCTGTTTTTATGGCTGGGATATGAAAGCACCGAA

**C** AAACCATACCGTTTTATCGTCCCGATTCTGTTTTTATGGCTGGGATATGAAAGCACCGAA

**11390**

**A** TCTTGCTGTATTCTACGGATACAACTTGGCGCAGCTTTCCTATCCGGCAAACGAAAATCC 125

**D** TCTTGCTGTATTCTACGGATACAACTTGGCGCAGCTTTCCTATCCGGCAAACGAAAATCC

**C** TCTTGCTGTATTCTACGGATACAACTTGGCGCAGCTTTCCTATCCGGCAAACGAAAATCC

**11390**

**A** CAATTCGTTCCTACATCTCACAGACGGCTCAAATCCGATTTGCTGCTTCATAGGCGAAGA 126

**D** CAACTCGTTCCTACATCTCGCAGACGGCTCAAATCCGATTTGCTGCTTCATAGGCGAAGA

**C** CAACCCGTTCCTACATCTCGCAGACGGCTCAAATCCGATTTGCTGCTTCATAGGCGAAGA

**11390**

**A** CGAGTATTTGCAAGCCAAACGGTTAGGTTTCAAAGAGTATGCCGATGCAAAAGCAAAGGC 127

**D** CGAGTATTTGCAAGCCAAACGGTTAGGTTTCAAAGAGTATGCCGATGCAAAAGCAAAGGC

**C** CGAGTATTTGCAAGCCAAACGGTTAGGTTTCAAAGAGTATGCCGATGCAAAAGCAAAGGC

**11390**

**A** TTTGGTTGATTTGGTTGAAATGGAGAAAAAATCCAAACAATCTGATTGAATCTTAAATTC 128

**D** TTTGGTTGATTTGGTTGAAATGGAGAAAAAATCCAAACAATCTGATTGAATCTTAAATTC

**C** TTTGGTTGATTTGGTTGAAATGGAGAAAAAATCCAAACAATCTGATTGAATCTTAAATTC

**A** AATAATAACGCGGTAGGGGGCTGGTCAAGCGAAGGTTCAGGCTCTGACTGAAATTTCGCT 129

**D** AATAATAACGCGGTAGGGGGCTGGTCAAGCGAAGGTTCAGGCTCTGACTGAAATTTCGCT

**C** AATAATAACGCGGTAGGGGGCTGGTCAAGCGAAGGTTCAGGCTCTGACTGAAATTTCGCT

**A** TGGACAGGGGGTGT 130

**D** TGGACAGGGGGTGT

**C** TGGACAGGGGGTGT
